# Enabling immune checkpoint blockade efficacy in T-lymphopenia by restoring CD8 T cell dynamics with IL-7 cytokine therapy

**DOI:** 10.3389/fimmu.2024.1477171

**Published:** 2024-12-16

**Authors:** Yeon-Woo Kang, Donghoon Choi, Dain Moon, Kun-Joo Lee, Youngsik Oh, Jaehyuk Yang, Siheon Jeong, Uni Park, Sun-Kyoung Im, Se Hwan Yang, Seung-Woo Lee

**Affiliations:** ^1^ Department of Life Sciences, Pohang University of Science and Technology, Pohang, Republic of Korea; ^2^ Research Institute of NeoImmuneTech, Inc., Pohang, Republic of Korea

**Keywords:** T-lymphopenia, treatment-related lymphopenia, interleukin-7, chemo/radiotherapy, tumor-infiltrating lymphocytes, tumor-reactive CD8 T cells, stem-like exhausted CD8 T cells

## Abstract

**Introduction:**

T-lymphopenia (TLP) is a frequently observed condition in cancer patients, often exacerbated by conventional chemo/radiotherapy, which impairs the efficacy of subsequent immune checkpoint blockade (ICB) therapy. This study aimed to understand the impact of TLP on ICB responsiveness and explore potential therapeutic strategies to enhance antitumor immunity.

**Methods:**

To investigate ICB responsiveness depending on the severity of TLP, first, we established TLP mouse models that mimic clinically observed mild and severe TLP through thymectomy and anti-Thy1-induced peripheral T cell depletion. T cell-replete mice and T-lymphopenic mice were inoculated with palpable or advanced tumors to evaluate the antitumor efficacy of anti-PD-1 therapy according to the severity of TLP. Additionally, by utilizing established murine models, we analyzed matched blood, tumor-draining lymph nodes (TDLNs), and tumor samples by flow cytometry to investigate the mechanisms by which ICB responsiveness is impaired under T-lymphopenic conditions. Finally, to evaluate the combination effect of anti-PD-1 and recombinant IL-7 cytokine therapy (rhIL-7-hyFc) in T-lymphopenic conditions, we administered anti-PD-1, rhIL-7-hyFc, or both to advanced tumor-bearing T-lymphopenic mice and subsequently analyzed tumor growth and survival rates.

**Results:**

Using mouse models mimicking clinical TLP, we observed that the antitumor efficacy of anti-PD-1 therapy was severely impaired in TLP, depending on the degree of TLP and the immunogenicity of the tumors. TLP mice showed a significant reduction in systemic CD8 T cells but stable intratumoral CD8 T cell numbers, suggesting maintained tumor infiltration despite systemic downregulation. Crucially, TLP led to a shift in the composition of tumor-infiltrating lymphocytes, with a decrease in PD-1^+^ tumor-reactive CD8 T cells and an increase in PD-1^−^ bystander cells. This reduction in PD-1^+^ cells was linked to impaired clonal expansion in tumor-draining lymph nodes. To counteract these effects, we introduced recombinant IL-7 cytokine therapy (rhIL-7-hyFc), which effectively restored systemic T cell counts, enhanced PD-1^+^ CD8 T cell proliferation within tumors, and increased the population of stem-like progenitor cells. The combination of rhIL-7-hyFc and anti-PD-1 therapy resulted in significant tumor regression and improved mouse survival.

**Discussion:**

Our findings highlight the critical role of IL-7 in reshaping the CD8 T cell landscape to improve ICB efficacy in TLP conditions, proposing a sequential therapeutic approach: conventional therapy to reduce tumor burden and enhance immunogenicity, followed by IL-7 therapy to restore and rejuvenate CD8 T cells, culminating in effective ICB treatment.

## 1 Introduction

Immune checkpoint blockades (ICB), such as anti-programmed cell death-1 (PD-1) and anti-programmed death ligand 1 (PD-L1), are widely used monoclonal antibodies to inhibit co-inhibitory signals by targeting the PD-1/PD-L1 axis (1, 2). While ICB therapy provides durable and long-term survival benefits over conventional therapies such as chemotherapy and radiotherapy (3), only a portion of solid cancer patients (~20%) experience the clinical benefits (4, 5). Therefore, a comprehensive analysis is necessary to identify factors limiting the responsiveness of cancer patients to improve the efficacy of ICB therapy.

CD8 T cells play a pivotal role in immune surveillance and antitumor immune responses (6). Indeed, a high abundance of CD8 tumor-infiltrating lymphocytes (TILs) correlates with favorable clinical outcomes in many cancer types (7, 8). However, persistent antigen exposure and immunosuppressive tumor microenvironment (TME) lead to CD8 T cell exhaustion, marked by increased expression of multiple co-inhibitory receptors, including PD-1, and progressive loss of effector functions (9, 10). Anti-PD-1 therapy aims to rejuvenate these exhausted PD-1^+^ CD8 T cells to enhance antitumor effects (11, 12). Thus, the presence of PD-1^+^ CD8 T cells in the TME is critical for the responsiveness to PD-1 blockade, as evidenced by clinical studies showing a strong correlation between PD-1^+^ CD8 TILs density and anti-PD-1 therapy response rates (13–16).

Lymphopenia is defined as a reduction in the blood lymphocyte count below the normal level (< 1000 cells/µl). In healthy humans, T cells constitute approximately 70% of peripheral blood lymphocytes, with B and NK cells accounting for 20% and 10%, respectively. Thus, severe and persistent lymphopenia often primarily manifests as T-cell lymphopenia (T-lymphopenia, TLP). TLP can occur in many cancer patients independently of prior treatment (17–21). Notably, due to the high radiosensitivity of lymphocytes, severe TLP is caused by chemo/radiotherapy (22), which is a standard of care treatment for solid cancer patients and occurs in approximately 40 to 90% of cancer patients with many different cancer types, including esophageal cancer, non–small cell lung cancer (NSCLC), and glioblastoma (GBM) (23–25). Furthermore, many studies have reported that moderate to severe TLP is associated with worse overall survival (24–28). More importantly, accumulating evidence suggests that cancer patients with lymphopenia respond poorly to ICB therapy (29–34). However, immunological mechanisms by which TLP contributes to the response to ICB therapy remain poorly elucidated. To develop a therapeutic strategy to further boost the efficacy of ICB therapy in cancer patients with TLP, a detailed analysis is essential to identify the underlying mechanisms responsible for low responsiveness to ICB therapy under T-lymphopenic conditions.

Interleukin−7 (IL−7), a member of the common γ-chain (γc) cytokines, is required for T cell development, survival, and homeostasis of mature T cells (35). Especially, IL-7 plays a crucial role in increasing T cell numbers during lymphopenic conditions by promoting homeostatic proliferation (36). Due to its advantageous effect as a T cell amplifier, exogenous IL-7 therapy has been extensively utilized in clinical trials to overcome TLP caused by viral infection (HIV, COVID-19, progressive multifocal leukoencephalopathy), idiopathic CD4 lymphocytopenia, and sepsis (NCT0047732, NCT02797431, NCT00839436, NCT03821038) (37–44). However, clinical use of IL-7 has been limited by its short *in vivo* half-life. To address this limitation, a hybrid Fc-fused recombinant human IL-7 (rhIL-7-hyFc, efineptakin alfa, NT-I7), a long-acting form of recombinant IL-7 fused with a stable hyFc fragment, was developed (45). Indeed, clinical studies reported a single dose of rhIL-7-hyFc induces sustained expansion of T cells in peripheral blood mononuclear cells (PBMCs) from healthy donors without adverse effects (NCT02860715) (46, 47). Moreover, a preclinical study revealed that rhIL-7-hyFc systemically expands CD8 T cells as well as enhances their tumor infiltration and significantly increases the number of PD-1^+^ CD8 TILs (48). The clinical and preclinical studies suggest the potential of rhIL-7-hyFc as a combination therapeutic drug with ICB therapy, where the abundance of PD-1^+^ CD8 T cells in the TME is important for successful antitumor efficacy.

Here, for the first time, we show a clear relationship between ICB efficacy and TLP in our T-lymphopenic mouse models mimicking clinical TLP. Furthermore, by utilizing established murine models, we analyzed matched blood and tumor samples to investigate the mechanisms by which ICB responsiveness is impaired under T-lymphopenic conditions and revealed that, despite a marked reduction in systemic CD8 T cells, a comparable intratumoral CD8 T cell count is maintained. Instead, a substantial decrease in PD-1^+^ tumor-reactive CD8 TILs, responders to PD-1 blockade, was observed in T-lymphopenic environments. Therefore, we propose rhIL-7-hyFc, a T cell amplifier, as a promising candidate to rescue a compromised antitumor efficacy of PD-1 blockade by restoring the number of PD-1^+^ tumor-reactive CD8 TILs in T-lymphopenic conditions.

## 2 Materials and methods

### 2.1 Mice

Seven-week-old female C57BL/6 (B6) mice were purchased from OrientBio Inc. (Seongnam‐si, Republic of Korea). All animals were maintained under specific pathogen-free (SPF) conditions in the animal facility at POSTECH Biotech Center. Animal care was performed in accordance with National Institutes of Health guidelines, and protocols were approved by the Institutional Animal Care and Use Committee (IACUC) of POSTECH.

### 2.2 Cell lines

MC38, B6 colon adenocarcinoma cell line, was kindly provided by Y.C. Sung (Genexine, Inc., Seongnam‐si, Republic of Korea), and B16F10, B6 skin melanoma cell line, was purchased from ATCC^®^ (Cat#CRL-6475™). MC38 and B16F10 were grown in Dulbecco’s modified Eagle’s Medium (DMEM) (Welgene, Cat#LM001-05) supplemented with 10% fetal bovine serum (FBS, GE Healthcare Life Sciences, Cat#SH30084.03) and 1× anti-anti (Gibco, Cat#15240). Cells were cultured in tissue-culture coated dishes (SPL), grown in at 37°C and 5% CO_2_ environment, and passaged when 70% confluent.

### 2.3 Mice thymectomy

Thymectomies were performed on eight-week-old mice under ketamine/xylazine anesthesia in aseptic conditions. After complete anesthesia, mice were fixed on the dissecting board, and their airways were opened by gently tilting the head backward using a rubber band. For sterilizing, the neck and upper chest area of the mouse were swabbed with 70% ethanol. A 1.5 to 2 cm midline longitudinal incision was made over the suprasternal notch. The first rib was cut using a scissor, and the chest was opened by extending forceps. After separating the strap muscles, the thymus was exposed, carefully gripped, and excised. The incision was promptly closed using an applier and clips for skin suturing. The entire procedure, from cutting the first rib to closing the skin, took less than 1 minute. Sham thymectomy involved a complete surgical procedure without thymic lobe removal. Thymectomized mice were allowed a six-week recovery period.

### 2.4 Tumor models

To establish mouse tumor models, the left flank or right thigh of the mice was shaved before inoculating tumor cells. For studies with a palpable tumor model, 1 × 10^5^ MC38 cells were subcutaneously (s.c.) inoculated into the left flank of mice, and tumor-bearing mice were randomized 7 days after tumor inoculation. For the establishment of an advanced tumor model, mice were inoculated s.c. with 1 × 10^5^ MC38 or B16F10 cells on the left flank or right thigh. MC38 tumor-bearing mice were randomized for even distribution between groups when the tumor reached 100 to 150 mm^3^ in the average volume. B16F10 tumor-bearing mice were randomized when the tumor reached 70 to 90 mm^3^ in the average volume. Tumors were measured three times a week using digital calipers. Tumor volume was calculated using the formula: length × width × width × 0.5, where length is the longest diameter, and width is the perpendicular diameter. Mice were euthanized when the longest diameter of the tumor exceeded 20 mm (for the MC38 tumor model) or the estimated tumor volume reached 2000 mm^3^ (for the B16F10 tumor model).

### 2.5 Drug treatment and local radiotherapy

STL mice were established by pretreating 200 µg of *InVivo*Mab anti-mouse Thy1.2 (clone 30H12, Bio-X-Cell, Cat#BE0066) T cell depleting antibody intraperitoneally (i.p.) 7 days before thymectomy. Local radiotherapy was delivered when the average tumor volume of each group reached as described above. Tumor irradiation was performed using an X-RAD 320 irradiator (Precision X-Ray Inc., North Branford, CT, USA) operated at 320 KV with a 1 mm cooper filter delivering a 3.5 Gy/min. Mice were anesthetized using i.p. injection of ketamine/xylazine. Mice were placed in lead shielding jigs and exposed tumors received 12 Gy irradiation. Cisplatin (4 mg/kg) was injected once i.p. into mice simultaneously with local irradiation. Mice were treated with either 10 mg/kg of *InVivo*MAb anti-mouse PD-1 (clone 29F.1A12, Bio-X-Cell, Cat#BE0273) or 10 mg/kg of *InVivo*MAb rat IgG2a isotype control (clone 2A3, Bio-X-Cell, Cat#BE0089) i.p. with a total of 3 times (3 days interval as described on study design from each experiment). rhIL-7-hyFc and its formulation buffer were kindly supplied by NeoImmuneTech, Inc. (Rockville, USA) via the Research Institute of NeoImmuneTech (Pohang, Republic of Korea). rhIL-7-hyFc (5 mg/kg) or formulation buffer was administered via s.c. route 2 days after local irradiation.

### 2.6 Preparation of single-cell suspensions

For single-cell suspension preparation, tumors were harvested and weighed at indicated time points. Tumors were chopped and digested with 400 Mandl units Collagenase D (Roche, Cat#11088882001) and 200 µg/ml DNase I (Roche, Cat#11284932001) for 30 min at 37°C while rotating. Following incubation, tumor pieces were mechanically dissociated through a 70-µm cell strainer. Spleen and LNs were also collected and passed through a 40-µm cell strainer to obtain single cells. Red blood cell (RBC) lysis was performed using RBC lysis buffer (Sigma, Cat#R7757). PBMC were separated from the whole blood by RBC depletion with RBC lysis buffer for 5 minutes. The total blood cell number was counted using the VetScan HM2 hematology analyzer (Abaxis). All single-cell suspensions were resuspended in complete Iscove’s Modified Dulbecco’s Medium (cIMDM) (Welgene, Cat#LM004-01) supplemented with 10% FBS, 1× anti-anti, and 55 µM 2-mercaptoethanol (Gibco, Cat#21985).

### 2.7 Flow cytometry

Mononuclear cells were isolated from tumors, LNs, spleen, and PB, and after achieving single cells, all single-cell suspensions were maintained in cIMDM for flow cytometry analysis. After washing with DPBS, cells were stained with Ghost Dye™ Violet 510 (Tonbo, Cat#13-0870) in PBS following the manufacturer’s protocol. Cells were washed with FACS buffer (0.5% FBS plus 0.09% sodium azide in DPBS) and blocked with Fc block (purified anti-mouse CD16/32 antibody, clone 93, BioLegend, Cat#101302) for 10 minutes at 4°C to reduce nonspecific immunofluorescent staining. Surface staining was performed for 30 minutes at 4°C in FACS buffer using the primary fluorophore-conjugated antibodies. For nuclear or cytoplasmic staining, cells were fixed and permeabilized with Foxp3/Transcription factor staining buffer set (eBioscience, Cat#00-5523-00) according to the manufacturer’s instruction followed by surface marker antibody staining. Flow cytometric analysis was performed on a CytoFLEX LX (Beckman Coulter) and analyzed by FlowJo software (Treestar Inc. Ashland, OR, USA). The following antibodies were used for the flow cytometry analysis: BioLegend or eBiosciences/Invitrogen or BD Biosciences; or from Cell Signaling: anti-CD4 (clone RM4-5), CD8α(clone 53-6.7), CD11b (clone M1/70), CD44 (clone IM7), CD45 (clone 30-F11), CD45R/B220 (clone RA3-6B2), CD62L (clone MEL-14), CD107a/LAMP-1 (clone 1D4B), CD127/IL-7Rα (clone A7R34), CD183/CXCR3 (clone CXCR3-173), CD195/CCR5 (clone HM-CCR5), CD279/PD-1 (clone RMP1-30), CD366/Tim-3 (clone RMT3-23), Ly6G (clone 1A8), and TCRβ (clone H57-597). Antibodies used for the intracellular staining include TCF1/TCF7 (clone C63D9), Foxp3 (clone FJK-16s), GzmB (clone GB11), and Ki-67 (clone SolA15). For detection of tumor antigen-specific CD8 T cells, cells were incubated with a PE-conjugated H-Kb/KWPWFTTL dextramer (Immudex, Virum, Denmark) for 30 min before surface staining.

### 2.8 Cytokine production analysis

For intracellular cytokine staining, cells from tumor tissues were restimulated with 20 ng/ml phorbol 12-myristate 13-acetate (PMA, Sigma, Cat#P8139) and 1 µg/ml ionomycin (Sigma, Cat#I0634) for 4 hours in the presence of GolgiStop (BD Biosciences, Cat#554724) and GolgiPlug (BD Biosciences, Cat#555029). After staining for the dead cells and surface marker, the cells were fixed and permeabilized with Cytofix/Cytoperm™ solution (BD Biosciences, Cat#554722, for cytokine staining) according to the manufacturer’s instructions. The following antibodies were used to stain intracellular cytokine: TNF-α (clone MP6-XT212) and IFN-γ (clone XMG1.2).

### 2.9 Statistical analysis

Statistical analyses were performed using Prism 10 software (GraphPad Software). Data were expressed as means with the SEM. Unpaired *t* test was used for the comparisons between two groups with independent samples. One-way or two-way analysis of variance (ANOVA) was used for > 2 comparison groups. *p* value of 0.05 and below was considered significant (*: *P* < 0.05; **: *P* < 0.01; ***: *P* < 0.001; and ****: *P* < 0.0001); ns, not significant.

## 3 Results

### 3.1 Establishment of T-lymphopenic mouse models

Lymphopenia can occur due to various factors; however, in cancer patients, it is primarily categorized into treatment-related lymphopenia and pretreatment lymphopenia. Treatment-related lymphopenia is caused by standard cancer therapies such as chemotherapy and radiation, which lead to a decrease in lymphocytes, whereas pretreatment lymphopenia naturally develops as the cancer progresses, independent of cancer treatment (**Supplementary Tables S1**, **S2**). Therefore, the lymphopenia experienced by many cancer patients often results from a combination of these two factors, with a notable persistence of lymphopenia for a significant period after cancer treatment, which profoundly affects the patient’s immune response. Given that approximately 70% of lymphocytes in human blood are T cells, which are essential for anticancer immune responses, it is reasonable that systemic lymphopenia leading to TLP correlates with the efficacy of ICB therapies, such as anti-PD-1 (**Supplementary Table S3**). Therefore, understanding the immunological mechanisms by which TLP contributes to the response to ICB therapy is essential. To address the challenge of overcoming TLP to enhance ICB efficacy, it is a priority to establish appropriate mouse models for experimentation.

**Figure 1:**
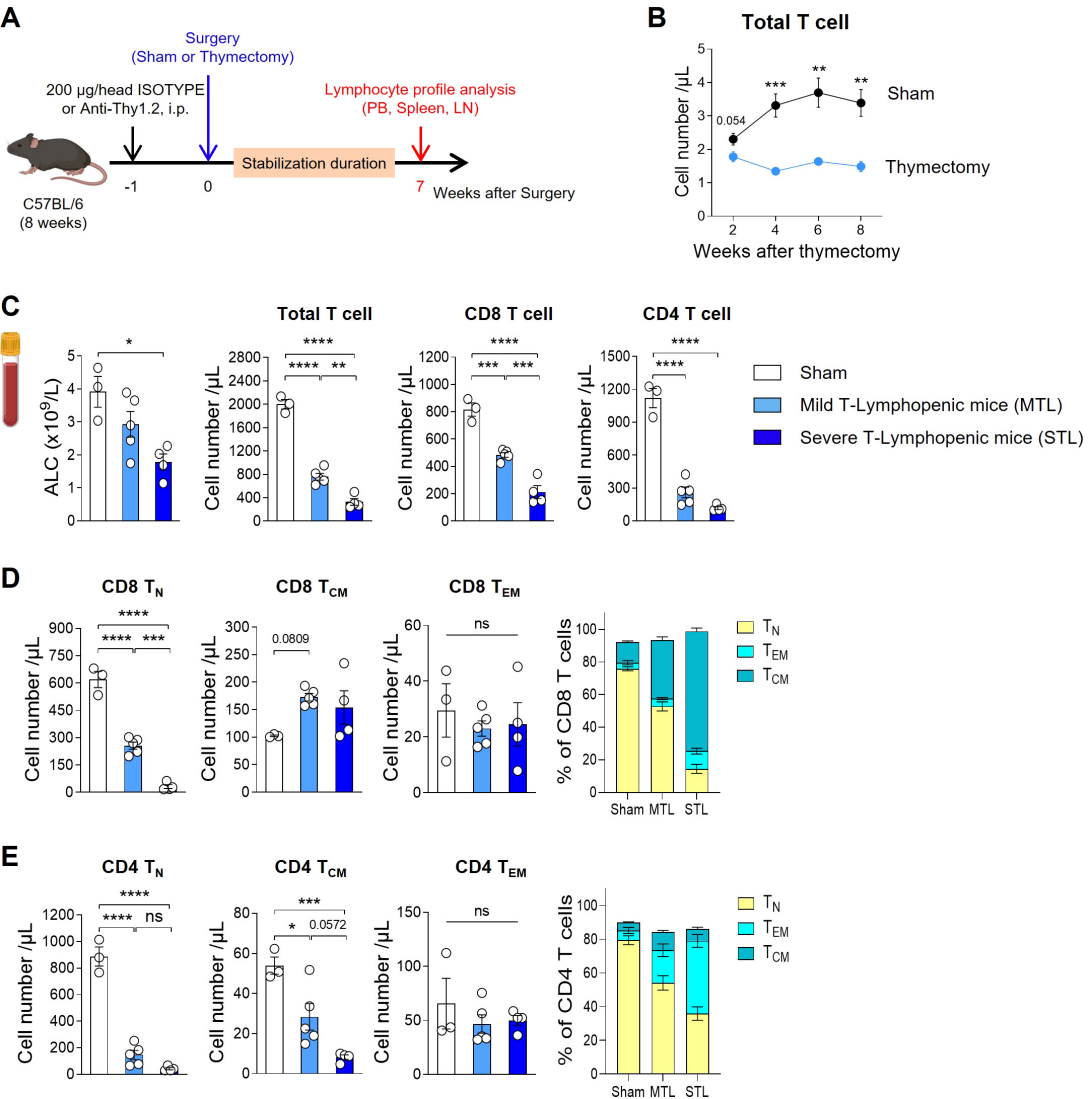
Establish a mouse model with T-lymphopenia. **(A)** Experimental scheme. Mild T-lymphopenic (MTL) mice were generated through thymectomy of 8-week-old C57BL/6 naïve mice. Severe T-lymphopenic (STL) mice were established by pretreating with T-cell depletion Ab (Thy1.2 mAb, 200 µg) through intraperitoneal (i.p.) injection one week before thymectomy. Thymectomized animals were allowed to recover for 6 weeks before peripheral lymphocyte profile analysis. **(B)** Peripheral blood (PB) was collected at 2, 4, 6, and 8 weeks post-sham or thymectomy surgery, and cells were analyzed by flow cytometry. Number of T cells at all time points studied (*n* = 5 per group). **(C–E)** Seven weeks after thymectomy, PB was collected from sham, MTL, and STL mice, and cells were analyzed by flow cytometry (*n* = 3-5 per group). **(C)** Number of total lymphocytes, total, CD8, and CD4 T cells. **(D, E)** Number and percentage of CD44^−^CD62L^+^ (Naïve T cells, T_N_), CD44^+^CD62L^+^ (Central memory T cells, T_CM_), and CD44^+^CD62L^−^ (Effector memory T cells, T_EM_) CD8 **(D)** and CD4 **(E)** T cells. All the data are shown as means ± SEM and representative of three independent experiments. ^*^ *P* < 0.05; ^**^ *P* < 0.01; ^***^ *P* < 0.001; and ^****^ *P* < 0.0001, unpaired two-tailed Student’s t-test at indicated time points **(B)** and one-way ANOVA with Bonferroni’s multiple comparison test for **(C–E)**. ns, not significant. All icons were “created with Biorender.com”.

As traditionally employed by many researchers, we induced TLP in young adult mice through thymectomy surgery (**Figure 1A**). Following the surgery, we tracked blood T cell counts and found that thymectomized mice exhibited a significant reduction in T cells compared to sham-operated mice starting at 4 weeks post-surgery, with chronic TLP persisting until 8 weeks (**Figure 1B**). Although B cell production in the bone marrow remained normal, resulting in only a slight reduction in the absolute lymphocyte count (ALC) compared to sham mice, there was a clear reduction in the T cell compartment. Notably, the CD4 subset showed a more pronounced decrease than the CD8 subset (**Figure 1C**).

Thymectomized mice displayed about 40% of the blood T cell levels seen in sham mice, a condition maintained by homeostatic proliferation of existing peripheral T cells even in the absence of thymic output. To develop a more severe model of TLP, we adopted a strategy of administering an antibody against Thy-1 (CD90), a pan-T cell marker, one week prior to performing thymectomy, thereby reducing the number of peripheral T cells (**Figure 1A**). After a 7-week stabilization period, designed to allow the effects of treatment to normalize, we analyzed T cell profiles in the peripheral blood (PB), spleen, and lymph nodes (LN). This approach enabled us to produce T-lymphopenic mice with less than 20% of the blood T cell levels found in sham-operated mice (**Figure 1C**). In humans, mild lymphopenia (grades 1 and 2) is defined as ALCs ranging from the lower limit of normal to 500 cells/µL, representing a reduction to less than half of the normal ALC. Severe lymphopenia (grades 3 and 4) is characterized by ALCs of less than 500 cells/µL, indicating a reduction to less than one-third of the normal ALC (CTCAE ver.5.0). Given that the reduction in T cells observed in our T-lymphopenic mouse models parallels the grading seen in humans, we have designated these models as mild and severe T-lymphopenic (MTL and STL) mice, respectively.

In our T-lymphopenic mouse models, significant changes were observed in T cell composition as expected. In sham mice, naïve cells comprised approximately 80% of total T cells, similar to normal mice, with the remainder being central memory (CM) and effector memory (EM) T cells. However, in MTL mice, there was a decrease in the naïve subset and an increase in memory subsets, a pattern that was more pronounced in STL mice (**Figures 1D, E**). This trend was similar across both CD8 and CD4 subsets, with CD8 cells showing a greater shift towards the CM subset, while CD4 cells exhibited a more substantial increase in the EM subset. When converted to absolute cell numbers, a consistent decline in naive T cells was observed in both subsets, although the reduction in memory cells was relatively less pronounced (**Figures 1D, E**). The gradual decrease in T cell numbers and changes in composition observed in MTL and STL mice were similarly detected in secondary lymphoid organs, including the spleen and LNs (**Supplementary Figures S1A, B**), as well as in PB. For Foxp3^+^ regulatory T cells (T_reg_), an increase in percentage but a decrease in absolute number was noted, mirroring the trends seen in CD4 memory cells in T-lymphopenic mice (**Supplementary Figure S1C**).

### 3.2 T-lymphopenia determines ICB responsiveness depending on tumor immunogenicity

**Figure 2:**
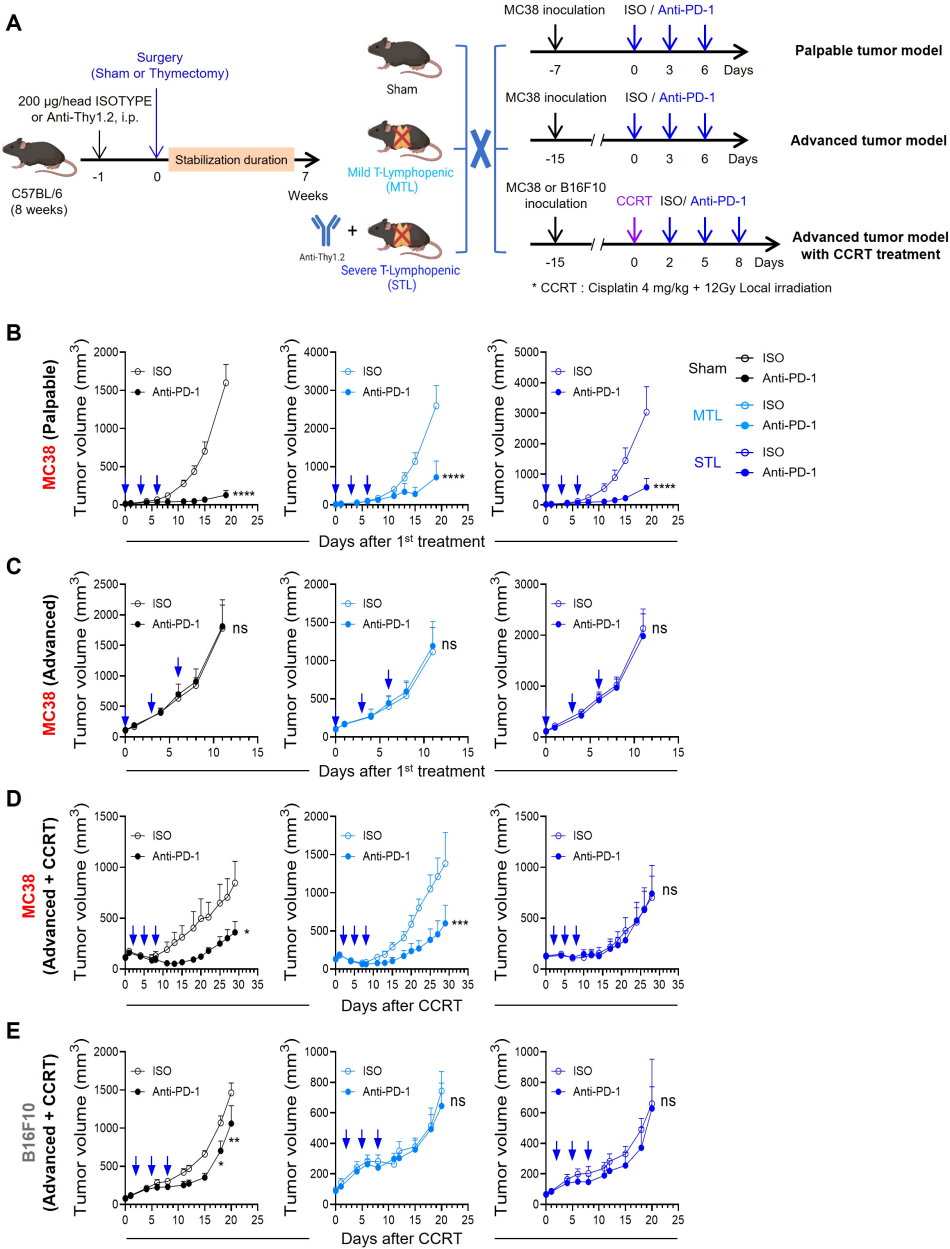
Antitumor efficacy of PD-1 blockade depends on tumor immunogenicity and T-lymphopenia severity. **(A)** Experimental scheme. Sham, MTL, and STL mouse models were established, and the antitumor efficacy of PD-1 blockade was evaluated in each mouse model in different tumor models. For studies with a palpable tumor model, 1 × 10^5^ MC38 cells were subcutaneously (s.c.) inoculated into the left flank of sham (*n* = 7-8 per group), MTL (*n* = 7 per group), and STL (*n* = 9 per group) mice. Seven days after tumor inoculation, 10 mg/kg of anti-PD-1 or rat IgG2b isotype control was administered i.p. with a total of 3 times every 3 days. For studies with an advanced tumor model, 1 × 10^5^ MC38 cells were s.c. inoculated into the left flank of sham (*n* = 9 per group), MTL (*n* = 5-6 per group), and STL (*n* = 8 per group) mice. When the average tumor volume reached 100 to 150 mm^3^, 10 mg/kg of anti-PD-1 or rat IgG2b isotype control was administered a total of 3 times every 3 days. For studies with an advanced tumor model with concurrent chemoradiotherapy (CCRT), 1 × 10^5^ MC38 or B16F10 cells were s.c. inoculated into the right thigh of sham (*n* = 7-8 per group), MTL (*n* = 5-6 per group), and STL (*n* = 9 per group) mice. An advanced tumor model was established when the MC38 or B16F10 tumor reached an average volume of 100 to 150 mm^3^ or 70 to 90 mm^3^, respectively. When the average tumor volume was reached at the indicated average tumor volume of each tumor model, CCRT, involving local radiotherapy (12 Gy) and i.p. injection of cisplatin (4 mg/kg), was administered. Mice were treated with 10 mg/kg of anti-PD-1 or rat IgG2b isotype control i.p. with a total of 3 times every 3 days from 2 days after CCRT administration. **(B–E)** Tumor growth curves in mice with palpable MC38 tumors **(B)**, advanced MC38 tumors **(C)**, and CCRT-treated advanced tumors: MC38 **(D)** and B16F10 **(E)**. For each type, curves are shown for sham (left), MTL (middle), and STL (right) mice. All the data are represented as means ± SEM and representative of two independent experiments. ^*^ *P* < 0.05; ^**^ *P* < 0.01; ^***^ *P* < 0.001, and ****P < 0.0001, two-way ANOVA with Bonferroni posttests **(B–E)**. All icons were “created with Biorender.com”.

Subsequently, we evaluated the antitumor efficacy of anti-PD-1 therapy in T-lymphopenic mouse models. Considering the immunocompromised state of T-lymphopenic mice, we opted for the MC38 colon carcinoma graft model, known for its immunogenicity and responsiveness to anti-PD-1 treatment (49, 50). MC38 tumor cells were inoculated, and treatment with anti-PD-1 was initiated seven days later when tumors became palpable. The therapy was administered every three days, totaling three doses (**Figure 2A**, top). Under these protocols, sham mice exhibited significant antitumor responses. Notably, despite their T-lymphopenic condition, both MTL and STL mice also displayed antitumor responses similar to those of sham mice (**Figure 2B**).

Next, we tested an advanced tumor model by inoculating MC38 cells and administering anti-PD-1 three times once the average tumor size reached approximately 100-150 mm^3^ (**Figure 2A**, middle). As previously reported, even the highly immunogenic MC38 tumors posed a significant challenge for treatment with anti-PD-1 therapy alone when the tumor burden is large (51, 52). Similarly, no antitumor efficacy was observed in either sham or T-lymphopenic mice harboring advanced MC38 tumors (**Figure 2C**). Therefore, we concluded that both the palpable and advanced MC38 models present challenges in assessing the responsiveness to ICB in the context of TLP, due to either excessively high or low tumor immunogenicity.

Consequently, we decided to enhance the immunogenicity of MC38 advanced tumors through anticancer treatment. Noting that clinical studies have shown lymphopenia to diminish responsiveness to ICB, we aimed to establish tumor conditions mimicking potential clinical scenarios. Thus, we chose concurrent chemoradiotherapy (CCRT), often used before or in conjunction with ICB in many cancer patients (53). This involved simultaneous tumor-localized irradiation (12 Gy) and intraperitoneal injection of cisplatin (4 mg/kg) on advanced MC38 tumors, followed by three doses of anti-PD-1 as part of our comprehensive treatment regimen (**Figure 2A**, bottom). In sham mice, CCRT temporarily slowed the growth of advanced tumors; however, growth resumed thereafter. Remarkably, subsequent treatment with anti-PD-1 significantly inhibited tumor growth. Under these conditions, the antitumor effect of anti-PD-1 was similar in MTL mice to that observed in sham mice, but was completely absent in STL mice (**Figure 2D**). These results suggest that the severity of TLP may determine ICB responsiveness when the TME is altered by conventional cancer therapies such as CCRT. Additionally, we tested the antitumor efficacy of the CCRT and anti-PD-1 combination in the B16F10 melanoma model, which is poorly immunogenic (50), similar to the advanced tumor setting. While the antitumor efficacy was statistically significant in sham mice, albeit lower than in the MC38 model, no antitumor response was observed in either MTL or STL mice (**Figure 2E**). This indicates that the impact of TLP on ICB responsiveness is more pronounced in conditions of low tumor intrinsic immunogenicity. In summary, our T-lymphopenic mouse models and experimental tumor conditions enable comparative studies of changes in ICB responsiveness due to TLP, closely resembling clinical settings.

### 3.3 Impact of T-lymphopenia on CD8 T cell dynamics in tumor microenvironments

**Figure 3:**
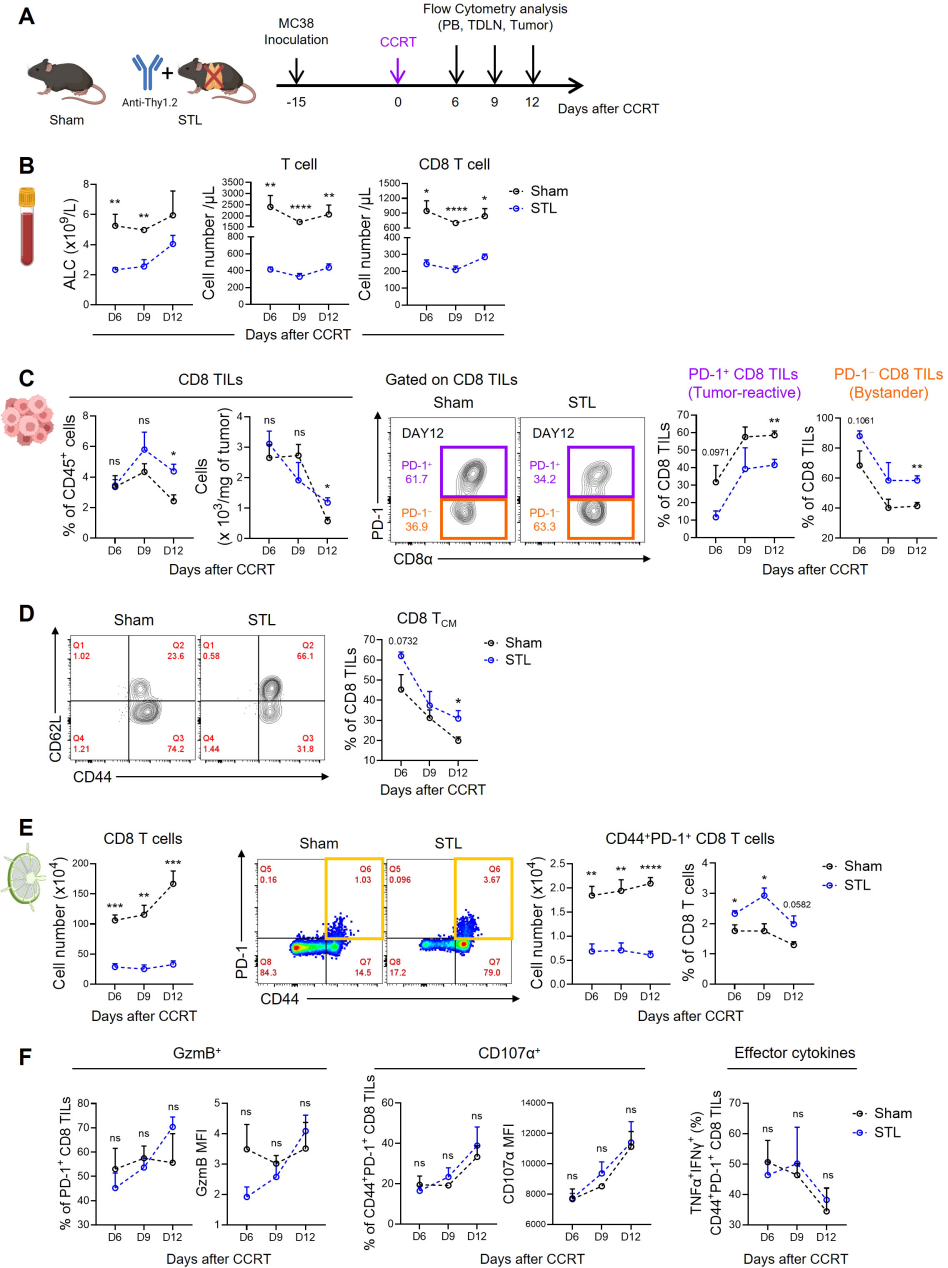
Impact of T-lymphopenia on the dynamics of CD8 T cells. **(A)** Experimental scheme. Advanced MC38 tumor-bearing sham (*n* = 4 per group) and STL (*n* = 4 per group) mice were administered CCRT (D0). PB, TDLNs, and tumor tissues from sham and STL mice were harvested on 6, 9, and 12 days post-CCRT, and immune cells were analyzed by flow cytometry at indicated days (D6, D9, and D12). **(B)** Absolute numbers of total lymphocytes (left), T cells (middle), and CD8 T cells (right) in PB. **(C)** Percentage and cell count per milligram of the CD8 TILs. Representative flow cytometry plots for PD-1^+^ tumor-reactive (purple) and PD-1^−^ bystander (orange) cells among CD8 TILs of sham and STL mice at day 12. PD-1^+^ and PD-1^−^ cells were identified through additional staining with a PD-1 isotype control or by using the spleen as an internal control. Percentage of PD-1^+^ and PD-1^−^ cells among CD8 TILs of sham and STL mice at indicated days. **(D)** Representative flow cytometry plots and percentage of T_CM_ cells among CD8 TILs. **(E)** Absolute numbers and percentage of CD8 T cells or activated CD44^+^PD-1^+^ CD8 T cells in TDLNs. Representative flow cytometry plots displaying the percentage of CD44^+^PD-1^+^ CD8 T cells among CD8 T cells in TDLNs of sham and STL mice at day 9. **(F)** Flow cytometric analysis of GzmB (left) and CD107α (middle) expression among PD-1^+^ CD8 TILs. TNF-α^+^IFN-γ^+^ cells (right) among PD-1^+^ CD8 TILs were analyzed after *ex vivo* stimulation with PMA plus ionomycin. MFI; Mean Fluorescence Intensity. All the data are represented as means ± SEM and representative of two independent experiments. ^*^ *P* < 0.05; ^**^ *P* < 0.01; ^***^ *P* < 0.001; and ^****^ *P* < 0.0001, unpaired two-tailed Student’s t-test at the same time point **(B–F)**. All icons were “created with Biorender.com”.

The immunological properties of the TME are shaped by the contributions of various immune and non-immune cells, which can create either an immunostimulatory or immunosuppressive environment. However, the success of immunotherapies like ICB can largely be attributed to the quantity and functionality of tumor-killing CD8 T cells. Therefore, we investigated changes in CD8 TILs under conditions where TLP is a significant factor affecting ICB responsiveness, specifically in STL mice with advanced MC38 tumors treated with CCRT (**Figure 3A**). Initially, to confirm the persistence of TLP under these conditions, we measured CD8 T cells in the blood. The results showed a significant reduction in both ALC and total T cell numbers in STL mice, with CD8 T cells decreased to about one-third of those in sham mice, indicating severe TLP (**Figure 3B**). Surprisingly, when examining the distribution of CD8 T cells within the tumors, we found that their numbers in STL mice did not decrease compared to sham mice (**Figure 3C**). This suggests that despite the reduction of CD8 T cells in the blood, a comparable number of CD8 TILs had accumulated in the tumors of STL mice.

Next, we assessed the composition of CD8 TILs under conditions of TLP. Preliminary studies categorized CD8 TILs in MC38 tumors based on PD-1 expression into PD-1^+^ tumor-reactive and PD-1^−^ bystander cells (54). Notably, in STL mice compared to sham mice, there was a decrease in PD-1^+^ CD8 TILs and an increase in PD-1^−^ TILs (**Figure 3C**; **Supplementary Figure S2A**). Furthermore, the majority of PD-1^−^ cells exhibited a CD44^+^CD62L^+^ T_CM_ phenotype (54), which was more prevalent in the tumors of STL mice (**Figure 3D**). According to the tumor-immunity cycle, PD-1^+^ tumor-reactive CD8 T cells are initially primed in tumor-draining lymph nodes (TDLNs) by antigens, migrate through the bloodstream to the tumor, and undergo further differentiation (55). To investigate whether the decrease in PD-1^+^ CD8 TILs in STL mice was due to compromised priming and activation, we analyzed CD8 T cells in the TDLNs. While the frequency of CD44^+^PD-1^+^ CD8 T cells in TDLNs of STL mice was similar to or slightly higher than in sham mice, their absolute numbers were significantly reduced (**Figure 3E**). This indicates that although early activation of CD8 T cells in TDLNs occurs unimpeded in STL mice, the overall decrease in CD8 T cell numbers limits the efficient clonal amplification of tumor-reactive PD-1^+^ CD8 T cells.

Indeed, in sham mice, strong immunogenic cell death (ICD) driven by CCRT released tumor antigens into the TDLNs, progressively amplifying CD8 T cell numbers over time. However, in STL mice, no increase in CD8 T cells was observed in the TDLNs (**Figure 3E**). This raises the question: how can the increased presence of PD-1^−^ CD8 TILs in the tumors of STL mice be explained? We found clues in the elevated expression of key chemokine receptors, specifically CXCR3 and CCR5, which facilitate tumor infiltration (56, 57). These receptors were upregulated on CD8 T_CM_ cells in the TDLNs and blood of STL mice compared to their sham counterparts (**Supplementary Figures S3A, B**). Lastly, we examined whether the functionality of PD-1^+^ tumor-reactive CD8 TILs in STL mice was altered. Expression levels of granzyme B and CD107α, which are critical for the cytolytic function of PD-1^+^ CD8 TILs, showed no differences between STL and sham mice. Similarly, the profile of effector cytokines secreted after stimulation was comparable between the two groups (**Figure 3F**). Therefore, although the number of PD-1^+^ tumor-reactive CD8 TILs in STL mice is reduced, their functional capacity remains intact.

Summarizing our findings, TLP leads to a significant reduction in the clonal expansion of tumor-reactive CD8 T cells within the TDLNs, consequently resulting in a marked decrease in PD-1^+^ CD8 T cells within the tumor. However, chronic TLP also induces homeostatic proliferation of CD8 T_CM_ cells, which express high levels of chemokine receptors that enhance tumor tropism, thereby increasing the number of PD-1^−^ CD8 T cells within the tumor. Therefore, despite the total number of CD8 TILs in T-lymphopenic mice not decreasing, the reduction in tumor-specific CD8 TILs appears to diminish responsiveness to ICB therapies, such as anti-PD-1.

### 3.4 IL-7 cytokine therapy enhances PD-1^+^ CD8 TILs in T-lymphopenic mice

Our results indicate that enhancing tumor immunogenicity and increasing the population of deficient PD-1^+^ CD8 TILs are essential for improving the efficacy of anti-PD-1 therapy in a T-lymphopenic environment. To address this, we have introduced recombinant IL-7 cytokine therapy, which has been validated for amplifying CD8 T cells in both mice and humans. We, along with others, have previously demonstrated that the long-acting cytokine Fc-fused rhIL-7 (rhIL-7-hyFc; epineptakin-alfa) can enhance antitumor functions when combined with chemo/radiotherapy or with ICB and T cell engager (TCE) immunotherapy (48, 54). The principal mechanism involves the amplification and functional modulation of CD8 T cells following the administration of rhIL-7-hyFc.

Initially, we investigated how rhIL-7-hyFc administration affects CD8 T cell dynamics in STL mice with CCRT-treated advanced MC38 tumors compared to sham mice. In previous studies, we performed a dose-escalation study of rhIL-7-hyFc in a palpable MC38 tumor model and observed significant antitumor efficacy across doses ranging from 1.25 to 10 mg/kg (48). Given these previous results, we have chosen 1.25 and 5 mg/kg in sham and STL mouse models with CCRT-treated advanced tumors (**Supplementary Figure S4A**). Both models exhibited dramatic tumor regression with a single dose of 5 mg/kg, making it the optimal choice for this research (**Supplementary Figure S4B**). After determining the optimal dose, we examined the effects of rhIL-7-hyFc on systemic CD8 T cell dynamics in each mouse model. Following rhIL-7-hyFc treatment, we observed a significant increase in circulating CD8 T cells in both groups (**Supplementary Figure S5A**). However, at the peak of T cell expansion, the increase in T cell numbers was approximately twice as high in sham mice compared to STL mice. Post-treatment, the CD8 T cell composition was predominantly T_CM_ cells; in sham mice, naïve T cells transitioned to T_CM_ cells, whereas in STL mice, the already elevated T_CM_ cells were further amplified (**Supplementary Figure S5B**). There was only a minimal increase in T_EM_ cells in both groups following rhIL-7-hyFc treatment.

**Figure 4:**
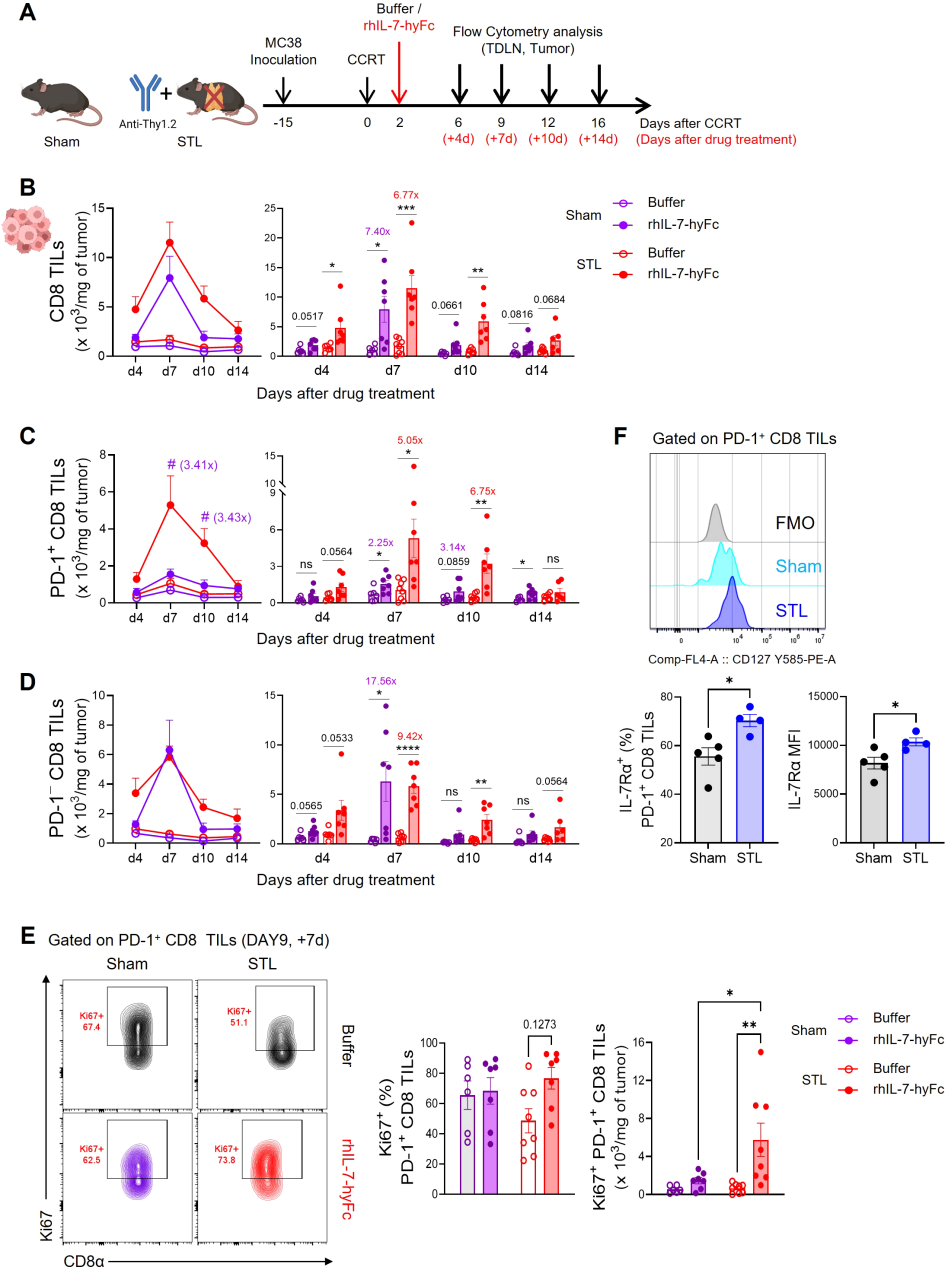
rhIL-7-hyFc treatment amplifies PD-1^+^ tumor-reactive CD8 TILs in T-lymphopenic condition. **(A)** Experimental scheme. Advanced MC38 tumor-bearing sham and STL mice were administered CCRT and treated with 5 mg/kg of rhIL-7-hyFc or buffer via s.c. route 2 days after CCRT administration. Tumor tissues were harvested on 4, 7, 10, and 14 days after rhIL-7-hyFc or buffer treatment, and immune cells were analyzed by flow cytometry at indicated days. **(B–D)** Number of total **(B)**, PD-1^+^ **(C)**, and PD-1^−^ **(D)** CD8 TILs of sham and STL mice at indicated time points for indicated treatment groups. **(E)** Representative flow cytometry plots (top) displaying Ki67 expression in tumors for PD-1^+^ CD8 TILs of buffer or rhIL-7-hyFc-treated sham and STL mice. Bar graph (bottom) displaying Ki67 expression among PD-1^+^ CD8 TILs in buffer-treated versus rhIL-7-hyFc-treated sham and STL mice at day 9 (7 days after buffer or rhIL-7-hyFc treatment). **(F)** Representative histogram, percentage, and MFI displaying IL-7Rα expression among PD-1^+^ CD8 TILs of sham and STL mice at 6 days post-CCRT. All the data are represented as means ± SEM of pooled biologically independent samples from two independent experiments (*n* = 3-4 per group) for **(B–E)**. *^/#^ *P* < 0.05; ^**^ *P* < 0.01; ^***^ *P* < 0.001; and ^****^ *P* < 0.0001, unpaired two-tailed Student’s t-test at the same time point **(B–D, F)** and one-way ANOVA with Bonferroni’s multiple comparison test **(E)**. * Compares with buffer treated versus rhIL-7-hyFc-treated group within sham or STL mice **(B–D)**, # compares with rhIL-7-hyFc-treated sham mice versus rhIL-7-hyFc-treated STL mice **(C)**. ns, not significant. All icons were “created with Biorender.com”.

Subsequently, we investigated changes in CD8 T cell dynamics within tumors in STL mice followed by rhIL-7-hyFc administration (**Figure 4A**). Post-treatment, both sham and STL mice exhibited a significant increase in CD8 TILs (**Figure 4B**). However, by day 7, the peak of T cell expansion, STL mice had more CD8 TILs in their tumors than sham mice, a disparity that was even more pronounced during the contraction phase from days 10 to 14. This suggests that, unlike the amplification of circulating T cells, the increase in CD8 T cells within tumors was more significant in STL mice following rhIL-7-hyFc treatment. Intriguingly, upon analyzing the composition of CD8 TILs, rhIL-7-hyFc treatment led to a significantly higher increase in PD-1^+^ tumor-reactive CD8 TILs in STL mice compared to sham mice (**Figure 4C**). For PD-1^−^ bystander TILs, both STL and sham mice demonstrated a similar pattern of increase, yet a more pronounced rise was noted in the contraction phase in STL mice (**Figure 4D**). Previous studies in T cell replete normal mice have shown that rhIL-7-hyFc administration results in a higher increase of PD-1^−^ bystander CD8 T cells within tumors (48, 54). Therefore, the selective amplification of PD-1^+^ CD8 TILs in STL mice following rhIL-7-hyFc treatment can be seen as a characteristic outcome driven by changes in IL-7-responsive CD8 T cells in a T-lymphopenic environment.

Since the origin of PD-1^+^ CD8 T cells within tumors is from TDLNs, we investigated whether rhIL-7-hyFc treatment led to a greater increase in PD-1^+^ CD8 T cells in TDLNs in STL mice compared to sham mice. However, the results showed that the number of both total and CD44^+^PD-1^+^ CD8 T cells were not higher in STL mice compared to sham mice (**Supplementary Figure S6A**). We also examined the expression of chemokine receptors involved in tumor trafficking, CXCR3 and CCR5, in CD44^+^PD-1^+^ CD8 T cells from TDLNs. While rhIL-7-hyFc treatment increased chemokine receptor expression in CD44^+^PD-1^+^ CD8 T cells in both groups, the levels of expression were similar between them (**Supplementary Figure S6B**). Thus, it is difficult to conclude that rhIL-7-hyFc treatment increases PD-1^+^ cells in TDLNs or enhances tumor tropism in STL mice. We then examined whether the proliferation of PD-1^+^ CD8 TILs within the tumor was enhanced by rhIL-7-hyFc treatment. In sham mice, neither the frequency nor the number of Ki67^+^ cells increased following rhIL-7-hyFc treatment; however, in STL mice, there was a clear increase in IL-7-induced proliferation (**Figure 4E**). We observed higher IL-7Rα expression on PD-1^+^ CD8 TILs in STL mice compared to sham mice (**Figure 4F**), suggesting that this may result in more potent IL-7-induced proliferative signaling in STL mice.

### 3.5 IL-7 cytokine therapy rescues the antitumor response of ICB in T-lymphopenic mice

Recent single-cell transcriptomic studies have revealed that PD-1^+^ CD8 T cells within tumors comprise heterogeneous subsets (54, 58). These subsets can largely be distinguished by the mutually exclusive expression patterns of the TCF-1 transcription factor, associated with stem-like characteristics, and the TIM-3 surface molecule, a marker of T cell exhaustion. Specifically, there are TCF-1^+^TIM-3^−^ stem-like or progenitor exhausted T cells (T_SL_ or T_PEX_) and TCF-1^−^TIM-3^+^ terminally differentiated or exhausted cells (T_TD_ or T_EX_). The distinction of these CD8 subsets is crucial, reflecting not only phenotypic differences but also functional implications for antitumor immunity. T_SL_ cells exhibit high proliferative capacity but lower cytotoxic function, while T_TD_ cells possess high effector functions but diminished self-renewal activity. Importantly, anti-PD-1 therapy primarily targets T_SL_ cells, promoting their differentiation into T_TD_ cells, which enhances antitumor efficacy (58–60).

In STL mice, the administration of rhIL-7-hyFc led to a characteristic increase in PD-1^+^ CD8 TILs, prompting us to investigate changes in the subsets of PD-1^+^ cells. Our results showed that rhIL-7-hyFc treatment in STL mice increased T_SL_ cells and decreased T_TD_ cells within the PD-1^+^ CD8 TIL composition (**Supplementary Figure S7A**). In our experimental setup, a substantial fraction of the observed CD8 subset were TCF-1^−^TIM-3^−^ cells, identified as intermediate cells (T_INT_) transitioning from T_SL_ to T_TD_ cells (61). Treatment with rhIL-7-hyFc also increased the frequency of this subset, similar to that observed in T_SL_ cells. However, when analyzed in terms of absolute numbers of CD8 TILs, rhIL-7-hyFc treatment resulted in an increase across all subsets compared to the buffer control, with the most pronounced increase seen in the TCF-1^+^TIM-3^−^ T_SL_ subset (**Supplementary Figure S7B**). Therefore, it can be concluded that rhIL-7-hyFc administration in STL mice significantly enhanced the TIM-3^−^ stem-like or intermediate subset. These results suggest that rhIL-7-hyFc preferentially amplified T_SL_ cells, supporting previous findings that TCF-1^+^TIM-3^−^ T_SL_ cells express higher levels of IL-7Rα compared to TCF-1^−^TIM-3^+^ T_TD_ cells (58, 59).

**Figure 5:**
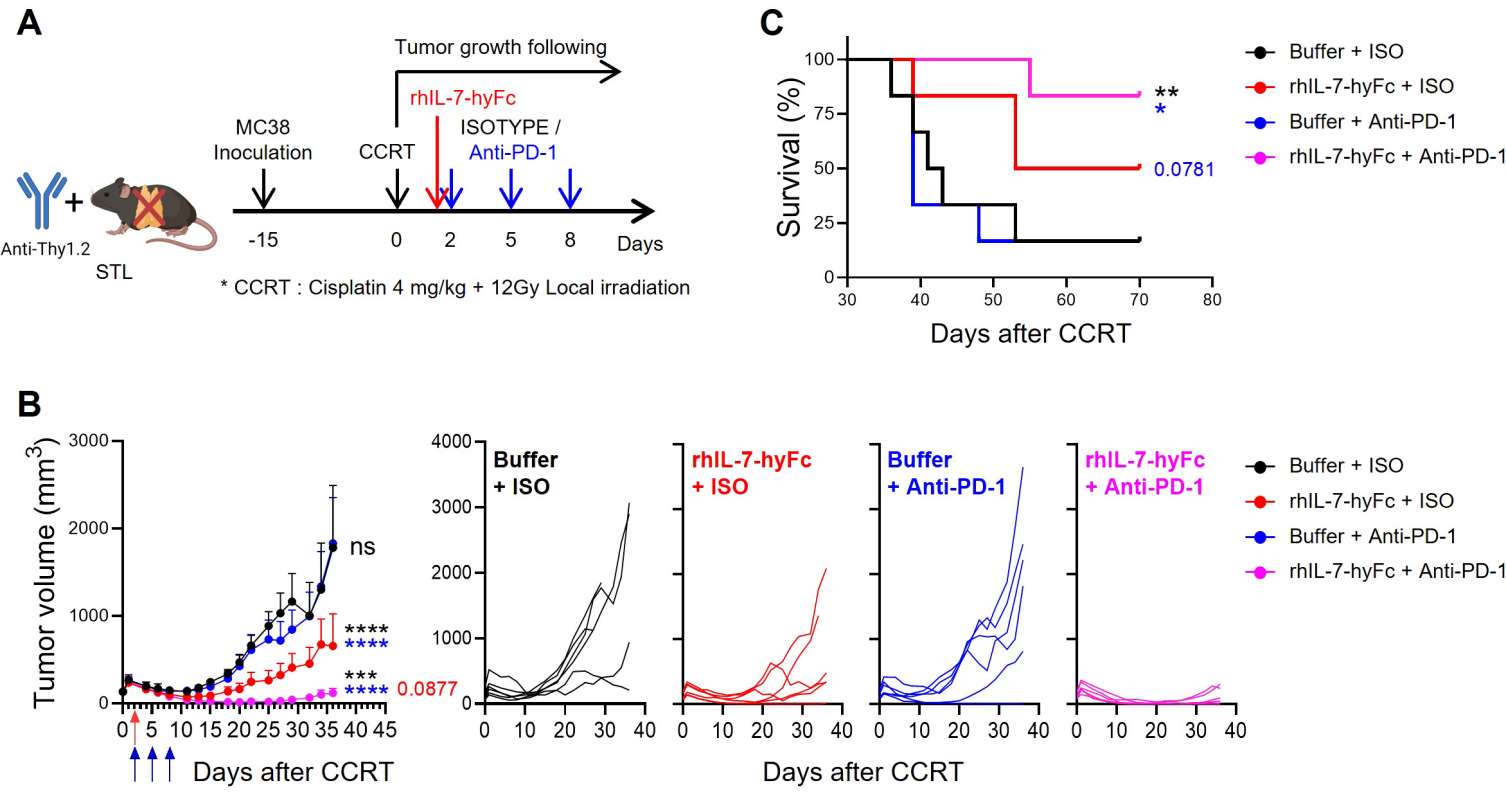
rhIL-7-hyFc optimizes the antitumor efficacy to anti-PD-1 therapy in T-lymphopenic mice. **(A)** Experimental scheme. Advanced MC38 tumor-bearing STL (*n* = 6 per group) mice were administered CCRT. rhIL-7-hyFc (5 mg/kg) or buffer was administered via s.c. route 2 days after CCRT administration. Rat IgG2b isotype (ISO) or anti-PD-1 (10 mg/kg) was administered i.p. with a total of 3 times every 3 days from 2 days after CCRT administration. **(B, C)** Tumor growth curves **(B)** and survival curves **(C)** in advanced MC38 tumor-bearing STL mice for indicated treatment groups. All the data are represented as means ± SEM and representative of three independent experiments. Statistical analysis was performed to compare the values between groups and the color of asterisks represents the comparison between corresponding color groups. ^*^ *P* < 0.05; ^**^ *P* < 0.01; ^***^ *P* < 0.001; and ^****^ *P* < 0.0001, two-way ANOVA with Bonferroni posttests **(B)** and log-rank (Mantel-Cox) test **(C)**. ns, not significant.

In summarizing our results, it appears that introducing rhIL-7-hyFc in a T-lymphopenic environment can induce an increase in PD-1^+^ CD8 TILs and enhance the antitumor efficacy of anti-PD-1 therapy. To experimentally validate this, we assessed tumor growth and mouse survival in STL mice with CCRT-treated advanced MC38 tumors, exploring the effects of additional treatments with either anti-PD-1 alone, rhIL-7-hyFc alone, or a combination of both. Consistent with the results shown in **Figure 2**, anti-PD-1 therapy alone did not inhibit the growth of advanced tumors in STL mice (**Figures 5A, B**). However, under the same conditions, rhIL-7-hyFc treatment alone demonstrated moderate antitumor efficacy, and the combination of rhIL-7-hyFc and anti-PD-1 dramatically inhibited tumor growth, nearly achieving complete regression. Similarly, long-term mouse survival was significantly enhanced only by the triple combination regimen of CCRT+rhIL-7-hyFc+anti-PD-1 in STL mice (**Figure 5C**). These findings suggest that IL-7 cytokine therapy can compensate for the deficiency of PD-1^+^ CD8 TILs caused by TLP and, by amplifying the TIM-3− subset sensitive to PD-1 blockade, optimize the antitumor efficacy of anti-PD-1 therapy.

### 3.6 Combination therapy with rhIL-7-hyFc and anti-PD-1 optimizes CD8 TIL potency in T-lymphopenic environments

**Figure 6:**
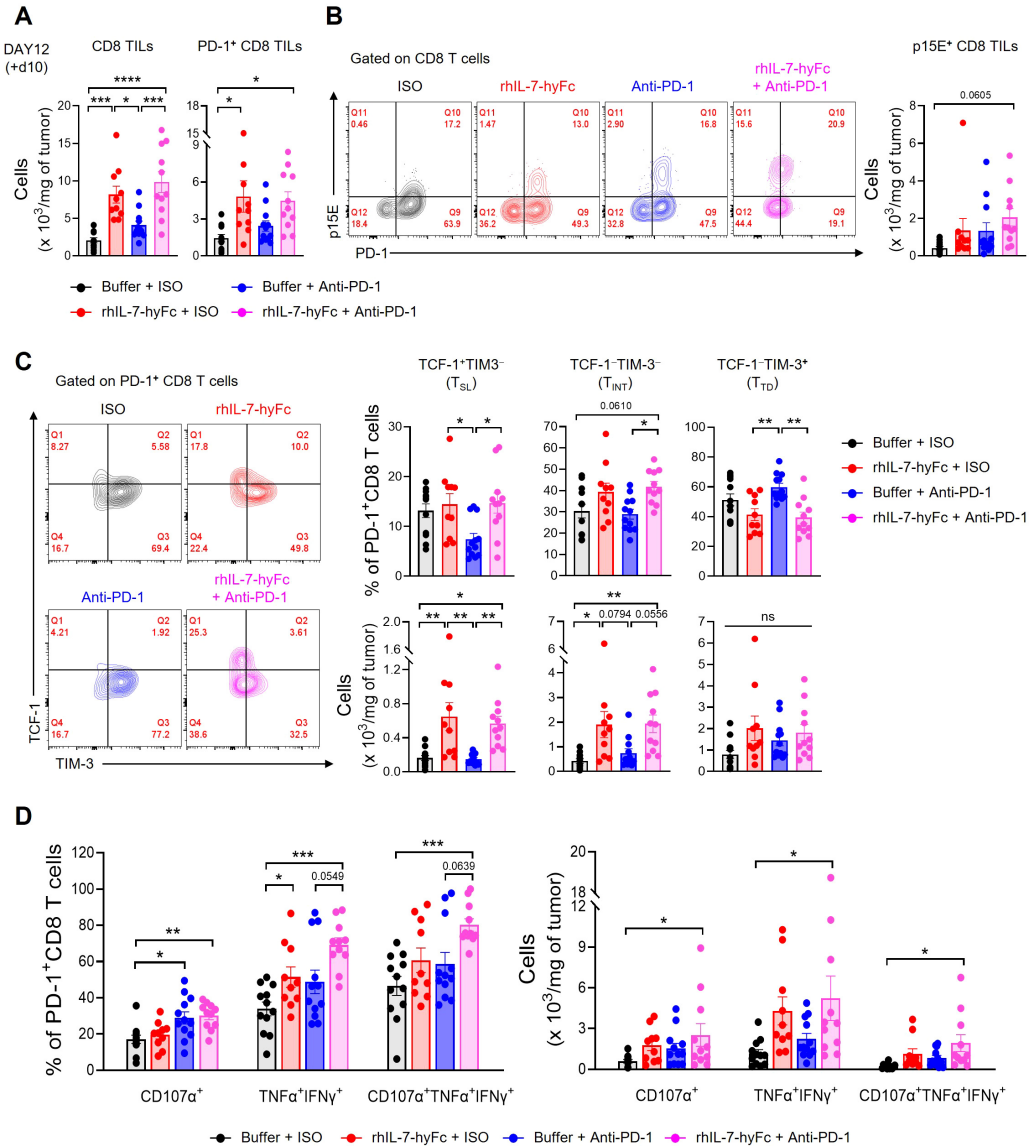
Combination of anti-PD-1 and rhIL-7-hyFc expands PD-1^+^ CD8 TILs with enhanced polyfunctionality. **(A–D)** Advanced MC38 tumor-bearing STL mice were treated, as shown in **Figure 5A**. Tumor tissues were harvested 10 days after rhIL-7-hyFc treatment and CD8 TILs were analyzed by flow cytometry. **(A)** Numbers of total (left) and PD-1^+^ (right) CD8 TILs for indicated treatment groups. **(B)** Representative flow cytometry plots (left) and absolute number (right) of p15E^+^ tumor antigen-specific CD8 TILs for indicated treatment groups. **(C)** Representative flow cytometry plots (left), percentage (right, top), and absolute numbers (right, bottom) of TCF-1^+^TIM-3^−^ (stem-like, T_SL_), TCF-1^−^TIM-3^−^ (intermediate, T_INT_), and TCF-1^−^TIM-3^+^ (terminally differentiated, T_TD_) PD-1^+^ CD8 TILs. **(D)** Percentage (left) and absolute number (right) of CD107α^+^, TNF-α^+^IFN-γ^+^, and CD107α^+^TNF-α^+^IFN-γ^+^ cells among PD-1^+^ CD8 TILs after *ex vivo* stimulation with PMA plus ionomycin. All the data are represented as means ± SEM of pooled biologically independent samples from two independent experiments (*n* = 5-7 per group) for **(A–D)**. ^*^ *P* < 0.05; ^**^ *P* < 0.01; ^***^ *P* < 0.001; and ^****^ *P* < 0.0001, one-way ANOVA with Bonferroni’s multiple comparison test. ns, not significant.

We next explored the mechanisms behind the synergistic antitumor immune response of rhIL-7-hyFc and anti-PD-1 combination therapy in a T-lymphopenic environment by analyzing CD8 TILs. We selected day 12 for the TIL analysis, a point before significant differences in tumor growth emerged among the groups, each of which had received CCRT followed by additional interventions with rhIL-7-hyFc and anti-PD-1. Initially, both total and PD-1^+^ CD8 TIL counts were significantly increased in the groups treated with rhIL-7-hyFc alone or in combination with anti-PD-1, compared to the CCRT-only treated control group (**Figure 6A**). While there was an increase in PD-1^+^ CD8 TILs following anti-PD-1 treatment, it was not statistically significant. Consistent with previous reports, a substantial fraction of the PD-1^+^ CD8 TILs in the MC38 tumor consisted of epitope-specific CD8 T cells derived from the p15E endogenous retrovirus (62, 63). The numbers of p15E^+^ CD8 TILs increased in all treatment groups relative to the control group, with the highest numbers observed in the rhIL-7-hyFc and anti-PD-1 combination group (**Figure 6B**).

Next, we analyzed the distribution of CD8 TIL subsets in STL mice following therapy. Examination of PD-1^+^ CD8 TIL frequency revealed that rhIL-7-hyFc administration increased TCF-1^+^TIM-3^−^ T_SL_ and TCF-1^−^TIM-3^−^ T_INT_ cells compared to controls, while TCF-1^−^TIM-3^+^ T_TD_ cells decreased (**Figure 6C**, top). In contrast, anti-PD-1 therapy resulted in an increase in TCF-1^−^TIM-3^+^ T_TD_ cells, a decrease in TCF-1^+^TIM-3^−^ T_SL_ cells, and no change in TCF-1^−^TIM-3^−^ T_INT_ cells. Consequently, rhIL-7-hyFc appears to enhance the composition of early to mid-stage progenitor subsets in the differentiation trajectory of CD8 TILs, whereas anti-PD-1 therapy promotes the prevalence of later-stage differentiated cells. However, the combined treatment of rhIL-7-hyFc and anti-PD-1 did not further alter the composition of CD8 TILs beyond the effects observed with rhIL-7-hyFc alone. This finding indicates that, like the overall CD8 TIL numbers, the subset distribution in STL mice is predominantly influenced by rhIL-7-hyFc. Furthermore, the absolute numbers of CD8 TIL subsets reveal that rhIL-7-hyFc predominantly increases T_SL_ and T_INT_ cells, whereas anti-PD-1 preferentially boosts T_TD_ cells (**Figure 6C**, bottom).

Lastly, we assessed the functionality of CD8 TILs. PD-1^+^ CD8 TILs expressing the cytotoxic molecule CD107α significantly increased in groups treated with either anti-PD-1 alone or in combination with rhIL-7-hyFc (**Figure 6D**). The absence of an increase in CD107α^+^ cells in the rhIL-7-hyFc-only group underscores the dependence of cytolytic function on anti-PD-1 therapy. Although all therapy groups showed enhanced secretion of the effector cytokines, TNF-α and IFN-γ, this effect was more pronounced with rhIL-7-hyFc. Intriguingly, the polyfunctional CD8 TILs, which display CD107α, TNF-α, and IFN-γ, were significantly more prevalent in the combination group, both in frequency and absolute number (**Figure 6D**). These findings highlight the complementary roles of rhIL-7-hyFc and anti-PD-1 therapy: rhIL-7-hyFc primarily augments effector cytokine secretion, while anti-PD-1 enhances cytolytic molecule expression, together promoting an increase in polyfunctional CD8 TILs in STL mice. Collectively, our results demonstrate that dual therapy with rhIL-7-hyFc and anti-PD-1 synergistically enhances both the quantity and polyfunctionality of CD8 TILs, thereby optimizing antitumor responses in T-lymphopenic environments.

## 4 Discussion

The Cancer-Immunity Cycle proposed by Chen and Mellman illustrates the various factors involved in antitumor immunity (55, 64). It also demonstrates that maintaining a sufficient number and function of CD8 CTLs throughout the entire T cell response, from priming to the effector phase, is crucial for a successful antitumor immune response. From this perspective, the observation that TLP in cancer patients hinders the proper development of an antitumor immunity is a reasonable conclusion. Numerous reports supporting the correlation between ICB responsiveness and TLP in various cancers further substantiate this notion (29–34, 65–68). Therefore, the aim of this study is to gain a fundamental understanding, through appropriate animal model experiments, of how TLP regulates antitumor immune responses, particularly the clonal expansion, tumor infiltration, and functions of CD8 T cells—areas that have been challenging to study in detail due to the limitations of clinical trials. Furthermore, this study aims to propose new immunotherapies that can significantly enhance ICB responsiveness by overcoming TLP.

In our study, the primary focus was to establish mouse models that demonstrate the relationship between TLP and the antitumor efficacy of ICB. Our TLP models have the following characteristics: First, the combination of thymectomy surgery and anti-Thy1-induced peripheral T cell depletion allows for the specific regulation of T cell numbers without disturbing other immune cells. This approach does not affect the development of B and myeloid-lineage cells, enabling the creation of mild and severe TLP similar to clinical grades. Second, considering that most cancer patients are aged (69), our model replicates the condition observed in many such patients, where thymic involution leads to a significant decrease in thymic output, compensated by homeostatic proliferation of T cells (70–72). Moreover, this model closely resembles the TLP experienced by many cancer patients during treatment, as it allows for the maintenance of chronic TLP throughout the entire experimental process (27, 73, 74). Third, our model closely mimics the changes in T cell composition seen in cancer patients; specifically, it shows a decrease in the naïve subset and an increase in the memory subset. Considering that most preclinical studies use young adult mice with a predominance of naïve cells, our model more accurately represents the T cell composition of aged patients undergoing anticancer therapy. Therefore, we believe that our TLP mouse model is significant as it simultaneously replicates both the reduction in T cell numbers and the changes in T cell composition observed in lymphopenic patients receiving clinical treatments (75).

Our animal model study suggests several critical points regarding the relationship between TLP and the antitumor efficacy of ICB. In the context of TLP, ICB responsiveness is influenced by tumor burden and the immunogenicity of the tumor. When the tumor burden is low (palpable model in this study), even severe TLP can still result in sufficient ICB responsiveness. This indicates that although the number of T cells is significantly reduced, the functionality of CD8 T cells remains largely intact, preventing a critical defect in the T cell response to tumors. Conversely, when the tumor burden is high (advanced model) but combined with treatments that can boost immunogenicity, such as CCRT, TLP becomes a decisive factor for ICB responsiveness. In this scenario, the immunogenicity of the tumor is determined by the intrinsic properties of the tumor cells combined with the immunosuppressive TME that develops as the tumor grows. Thus, in such cases, a sufficient number of T cells capable of responding to tumor antigens is necessary to induce an effective antitumor immune response via ICB. Additionally, the immunogenicity of tumors can be enhanced by cancer treatments. The CCRT we used is known to enhance tumor immunogenicity by releasing tumor antigens through immunogenic cell death and activating innate immunity pathways such as the cGAS-STING pathway (76, 77). However, our results indicate that even if the immunogenicity of the tumor is increased through treatments, the antitumor efficacy of ICB is limited if there are not enough CD8 T cells due to TLP. This limitation is particularly critical in cases where the intrinsic immunogenicity of the tumor is low, such as B16F10 compared to MC38, where TLP can act as a more significant factor.

The analysis of systemic versus tumoral CD8 T cell dynamics in the context of TLP offers intriguing insights. Contrary to expectations, despite a significant decrease in systemic CD8 T cells during TLP, the number of intratumoral CD8 T cells remained stable. This observation suggests that if continuous infiltration of T cells into the tumor is possible, the number of T cells within the tumor can be maintained, even with a reduction in systemic T cell frequency. However, a critical issue in TLP is the substantial alteration in the composition of CD8 T cells infiltrating the tumor. Specifically, there is a marked decrease in the influx of PD-1^+^ tumor-reactive CD8 T cells, which are replaced by PD-1^−^ bystander cells. Our study demonstrated that the primary cause for the reduction of PD-1^+^ CD8 T cells during TLP is the absolute decrease in the number of CD8 T cells within the TDLNs, where clonal amplification of CD8 T cells responding to tumor antigens is initiated. Consequently, the reduction of PD-1^+^ tumor-reactive CD8 T cells explains the decreased ICB responsiveness in the context of TLP.

To overcome this challenge, we demonstrated that recombinant IL-7 cytokine therapy could serve as an effective solution. Administration of rhIL-7-hyFc, a clinically validated T-cell amplifier, altered CD8 T cell dynamics under TLP conditions in the following ways. Firstly, it restored the reduced number of systemic T cells through the amplification of peripheral T cells. Notably, it increased the number of PD-1^+^ T cells in the TDLNs, likely by aiding the priming process of tumor antigens exposed through CCRT. A more significant finding was that rhIL-7-hyFc markedly increased the proliferation of PD-1^+^ CD8 T cells within the tumor. The proliferated PD-1^+^ cells predominantly belonged to the TIM-3− stem-like progenitor populations. The reason rhIL-7-hyFc acts primarily on this subset is twofold: stem-like populations express higher levels of IL-7Rα (58, 59), and IL-7R signaling, unlike that of the effector cytokine IL-2, mainly affects T cell persistence and homeostasis (78). This has critical implications for ICB efficacy since the major CD8 T cells targeted by anti-PD-1 are the PD-1^+^TIM-3− cells amplified by rhIL-7-hyFc (58–61, 79). Thus, the administration of rhIL-7-hyFc in the context of TLP not only restores T cell numbers but also modifies the composition of intratumoral CD8 T cells to enhance responsiveness to ICB. Furthermore, the addition of anti-PD-1 enhances the polyfunctionality of CD8 TILs, leading to more effective tumor control.

Extensive research is underway to apply rhIL-7-hyFc to cancer patients experiencing treatment-related lymphopenia (TRL), such as those with GBM, a prototypical immune-cold tumor. Standard treatments like radiotherapy (RT) and temozolomide (TMZ) often induce severe, prolonged lymphopenia, which affects approximately 40% of GBM patients and is associated with worse survival outcomes (27, 80). Given IL-7’s role in T cell homeostasis, IL-7-mediated immunotherapy may be particularly effective under lymphopenic conditions. Notably, Campian et al. showed that rhIL-7-hyFc administration under RT/TMZ-induced lymphopenia improved survival in GBM murine models by expanding CD8 TILs with enhanced effector function (81). These findings highlight rhIL-7-hyFc as a promising therapeutic strategy for GBM, currently being evaluated in a phase I/II trial (NCT03687957). Despite the promising therapeutic effects of rhIL-7-hyFc under TLP conditions, careful consideration is required for its clinical application. As rhIL-7-hyFc induces a robust expansion of effector T cells without expansion of Tregs, there exists a potential risk of autoimmune disease development in certain individuals (47, 82, 83). To address this concern, clinical trials should carefully select appropriate patient cohorts and conduct dose-escalation studies to optimize dosing regimens that maximize T cell amplification without adverse drug reactions and potential side effects. With these precautions, IL-7 therapy is expected to provide maximal therapeutic benefits while minimizing potential risks, making it a viable option for clinical use in cancer patients with TLP.

In summary, our study suggests that for effective ICB therapy in the context of TLP, it is crucial to first introduce anticancer treatments, like CCRT, that increase tumor immunogenicity by considering the tumor burden and inherent immunogenicity. Additionally, it proposes the necessity of therapies that amplify the reduced systemic T cells and induce a tumor-reactive composition of CD8 T cells within the tumor, ensuring an adequate stem-like subset highly responsive to anti-PD-1. Therefore, we propose that the most effective immunotherapy for TLP would involve a sequence starting with conventional therapy to reduce tumor burden and enhance immunogenicity, followed by the establishment of an appropriate CD8 T cell landscape within the tumor using rhIL-7-hyFc treatment, and finally, the application of ICB to achieve superior effector functions of CD8 T cells.

### 4.1 Limitation of the study

Although the TLP mouse model used in this study closely mimics the T cell dynamics observed in clinical patients compared to normal mice, it is challenging to fully capture the diverse aspects of TLP as it occurs in humans. Future research efforts should aim to incorporate these elements into the TLP model, particularly age-associated immunosenescence, to better represent the immune environment of many aged cancer patients. While this study primarily focused on the effects of rhIL-7-hyFc on CD8 T cells, it is likely that rhIL-7-hyFc also influences various immune cells, including CD4 T cells and myeloid cells within tumors. To fully elucidate the mechanisms underlying the antitumor responses mediated by rhIL-7-hyFc, future studies employing single-cell-based approaches to comprehensively analyze immune cell dynamics within the TME are necessary. In clinical settings, the correlation between TLP and anticancer treatment has primarily been explored through retrospective studies. Therefore, the most critical step forward is to conduct prospective clinical studies that longitudinally analyze CD8 T cell dynamics in TLP patients under conventional treatment followed by combination therapy with IL-7 and ICB, as suggested by our findings.

## Data Availability

The original contributions presented in the study are included in the article/**Supplementary Material**. Further inquiries can be directed to the corresponding author.
