# Supplementary material for: Enabling immune checkpoint blockade efficacy in T-lymphopenia by restoring CD8 T cell dynamics with IL-7 cytokine therapy
Source: Front Immunol. 2024 Dec 16;15:1477171. doi: 10.3389/fimmu.2024.1477171 (PMC11701376; doi:10.3389/fimmu.2024.1477171)
Supplement: Supplementary file 1 [file DataSheet1.docx]

**Supplementary Tables**

**Supplementary Table S1. Incidence rates of pre-treatment lymphopenia and the relationships between lymphopenia and survival after treatment in solid cancer patients.**

| Cancer Type | Definition of Lymphopenia | Incidence Rate (%) | Post-treatment | OS or PFS difference in months or rate  Lymphopenia vs Normal | Ref. |
| --- | --- | --- | --- | --- | --- |
| Esophagus | ALC < 1000 cells/μL | 16.6 | Surgery ±  CT or RT | 5yr CSS: 21.6% vs 43.8% (*p* = 0.004) | (1) |
|  |  | 19.0 | CT | OS: 8.2 vs 12.7 (*p* = 0.02) | (2) |
| Cervix | ALC < 1000 cells/μL | 15.0 | CCRT | OS: 10.6 vs 45 (*p* < 0.01) PFS: 4.8 vs 10.6 (*p* < 0.01) | (3) |
|  |  | 11.0 | CCRT | 5yr OS: 20% vs 61% (*p* < 0.001) | (4) |
| Metastatic breast carcinoma | ALC < 1000 cells/μL | 25.0 | CT (FEC) | OS: 9.5 vs 13.6 (*p* < 0.0001)  PFS: 6.8 vs 8.5 (*p* = 0.0001) | (5) |
|  | ALC < 700 cells/μL (Severe lymphopenia) | 22.3 | CT | OS: 6.6 vs 21.7 (*p* = 0.0093) | (6) |
| SCLC | ALC < 1000 cells/μL | 23.0 | CT ± RT | OS: 9.8 vs 12.0 (*p* = 0.013) | (7) |
| Colorectum | ALC < 1000 cells/μL | 19.1 | CT | OS: 19 vs 24 (*p* = 0.024)  PFS: 4 vs 7 (*p* = 0.033) | (8) |
| Sarcoma | ALC < 1000 cells/μL | 24.0 | CT (CYVADIC) | OS: 5.4 vs 9.6 (*p* = 0.0057) | (5) |
|  | ALC < 700 cells/μL | 17.0 | CT |  | (9) |

The survival rates compare post-treatment survival between cancer patients with or without pre-treatment lymphopenia.

Abbreviations: SCLC = small cell lung cancer; ALC = absolute lymphocyte count; CT = chemotherapy; RT = radiotherapy; CRT = chemoradiotherapy; CCRT = concurrent chemoradiotherapy; FEC = fluorouracil, epirubicin hydrochloride, and cyclophosphamide; CYVADIC = cyclophosphamide, vincristine, doxorubicin,and dacarbazine; CSS = cancer-specific survival; OS = overall survival; PFS = progression-free survival.

**Supplementary Table S2. Incidence rates of post-treatment lymphopenia and the relationships between lymphopenia and survival after treatment in solid cancer patients.**

| Cancer Type | Definition of Lymphopenia | Incidence  Rate (%) | | Post-treatment | OS or PFS difference in months or rate  Lymphopenia vs Normal | Ref. |
| --- | --- | --- | --- | --- | --- | --- |
|  |  | Grade 3-4 | Grade 4 |  |  |  |
| Esophagus | Grade 3: ALC 200–500 cells/μL Grade 4: ALC < 200 cells/μL | 91.0 | 39.0 | CRT | 5yr OS: 35.4% vs 51.8% (*p* < 0.001)  5yr PFS: 30.1% vs 40.7% (*p* = 0.002) | (10) |
|  |  | 88.3 | 23.6 | CRT | Low OS (*p* < 0.001) and PFS (*p* < 0.001) | (11) |
|  |  | 96.0 | 50.0 | CCRT | 5yr OS: 35.6% vs 61.6% (*p* = 0.023)  5yr PFS: 37.9% vs 48.1% (*p* = 0.032) | (12) |
|  |  | 86.0 | 27.0 | CRT | OS: 33.6 vs 60 (*p* = 0.027)  PFS: 13.2 vs 61.2 (*p* < 0.001) | (13) |
| NSCLC | ALC < 500 cells/μL after RT | 54.8 |  | CRT | OS: 23 vs 34 (*p* = 0.008)  PFS: 10 vs 13 (*p* = 0.003) | (14) |
|  |  | 49.0 |  | RT |  | (15) |
| SCLC | Minimum ALC < 297 cells/μL  or ALC < 698 cells/μL after RT  (1 month) | 35.6 |  | CCRT | OS: 19.3 vs 46.9 (*p* = 0.001) | (16) |
|  | ALC < 655 cells/μL after RT  (1 month)  or ALC < 1430 cells/μL after RT (3 months) | 61.1 |  | CT | OS: 18.1 vs 36 (*p* < 0.001)  PFS: 9.7 vs 26.2 (*p* < 0.001) | (17) |
| Head and Neck | ALC < 500 cells/μL after RT | 73.0 |  | RT | OS: HR; 2.94 | (18) |
| Liver | ALC < 500 cells/μL after RT | 87.0 |  | RT | OS: 13.6 vs 46.7 (*p* < 0.001) | (19) |
| Nasopharynx | Minimum ALC < 245 cells/μL | 48.6 |  | CRT | PFS: 35.2% vs 71.2% (*p* = 0.004) | (20) |
|  | Minimum ALC < 390 cells/μL  or ALC < 705 cells/μL after CCRT (3 months) | 48.0-71.0 |  | CCRT | OS: 69.4 vs 90.5 (*p* < 0.001)  PFS: 61.7 vs 79.8 (*p* < 0.001) | (21) |
| Brain | ALC < 500 cells/μL after RT | 37.0 |  | CT (TMZ) + RT | OS: 16 vs 18 (*p* = 0.02) | (22) |
|  | CD4 T cells < 200 or 300 cells/μL | 73.0 | 40.0 | CT (TMZ) + RT | OS: 13.1 vs 19.7 (*p* = 0.002) | (23) |
| Pancreas | ALC < 500 cells/μL after RT | 45.0 |  | CT (Gemcitabine/5-FU) + RT | OS: 14 vs 20 (*p* = 0.05) | (22) |
|  |  | 45.0 |  |  | OS: 8.7 vs 13.3 (*p* = 0.03) | (24) |
|  | Grade 3: ALC 200–500 cells/μL Grade 4: ALC < 200 cells/μL | 43.3 |  | CCRT | OS: 7.97 vs 19.83 (*p* < 0.001)  PFS: 5.13 vs 12.4(*p* < 0.001) | (25) |
| Cervix | ALC < 500 cells/μL after RT | 61.0 |  | CCRT | 5yr OS: 20% vs 61% (*p* < 0.001) | (4) |

The survival rates compare post-treatment survival between cancer patients with or without post-treatment lymphopenia.

Abbreviations: NSCLC = non-small cell lung cancer; SCLC = small cell lung cancer; ALC = absolute lymphocyte count; CT = chemotherapy; RT = radiotherapy; CRT = chemoradiotherapy; CCRT = concurrent chemoradiotherapy; OS = overall survival; PFS = progression-free survival; HR = hazard ratio; TMZ = temozolomide.

**Supplementary Table S3. Relationships between lymphocyte counts and clinical responses in solid cancer patients treated with PD-1 blockade.**

| Cancer Type | Definition of Lymphopenia | Chemotherapy/Radiotherapy (Prior treatment) | OS difference in months  Lymphopenia vs Normal | Ref. |
| --- | --- | --- | --- | --- |
| NSCLC | ALC < 1000 cells/μL | Last chemotherapy (cytotoxic drugs) : 84% of total patients. RT during immunotherapy : 24.6% of total patients. | 5.7 vs 12.1 (*p* < 0.001) | (26) |
|  | ALC < 500 cells/μL (Severe lymphopenia) | - RT delivered within 14 days of the start of ICB: 3% of patient  - RT delivered in the 30 days before start of ICB: 12% of patient  - RT delivered only before start of ICB  : 25% of patient  - RT delivered only after start of ICB  : 5% of patient  - RT delivered both before and after start of ICB: 18% of patient | 3.3 vs 8.2 (*p* = 0.008) | (27) |
|  | CD3^+^ lymphocytes < 1015 cells/μL |  | HR = 0.48 (*p* = 0.017) | (28) |
| Melanoma | ALC < 1000 cells/μL |  | 28.1 vs 36.8 (*p* = 0.01) | (29) |
| non-CRC  GI cancer | ALC < 1000 cells/μL |  | 6.23 vs 9.87 (*p* = 0.006) | (30) |
| Cancer Type | Definition of Lymphopenia | Chemotherapy/Radiotherapy (Prior treatment) | PFS difference in months Lymphopenia vs Normal | Ref. |
| NSCLC | ALC < 230 cells/μL (Severe lymphopenia) | 62% of patients received CCRT prior to the ICB therapy.  -Chemotherapy consisted of arboplatin/cisplatin plus paclitaxel or pemetrexed. - Radiation (to a total dose of at least 60 Gy) was delivered five times per week in fractions of 1.2 to 2.0 Gy each. | 17.1 vs 37.7 (*p* = 0.02) | (31) |
|  | ALC < 1000 cells/μL | Last chemotherapy (cytotoxic drugs) : 84% of total patients  RT during immunotherapy : 24.6% of total patients | 2.2 vs 5.9 (*p* < 0.001) | (26) |
|  | ALC < 500 cells/μL (Severe lymphopenia) | Types of concurrent Chemotherapy - Radiosensitizing: 40% of total patients - Full dose platinum: 38% of total patients Median total RT delivered: 63Gy | 7.23 vs 19 (*p* < 0.001) | (32) |
| Head and Neck | ALC < 600 cells/μL | 47.1% of patients received two or more lines of first-line CCRT prior to the ICB therapy while the remaining patients had 0 or 1 prior lines of CCRT. | 2 vs 4.7 (*p* < 0.05) | (33) |
|  | ALC < 770 cells/μL |  | 5.6 vs 3.1 (*p* = 0.002) | (34) |
| Esophageal | ALC < 500 cells/μL | 31.1% of patients received 2 or more lines of previous chemotherapy prior to ICB therapy. 42.5% of patients received RT before and during ICB therapy. | 4.8 vs 7.0 (*p* = 0.009) | (35) |
| Melanoma | ALC < 1000 cells/μL |  | 13.3 vs 16.9 (*p* = 0.025) | (29) |

Abbreviations: NSCLC = non-small cell lung cancer; non-CRC GI cancer = non-colorectal gastrointestinal cancer; PFS = progression-free survival; OS = overall survival; ALC = absolute lymphocyte count; RT = radiotherapy; CCRT = concurrent chemoradiotherapy.

**
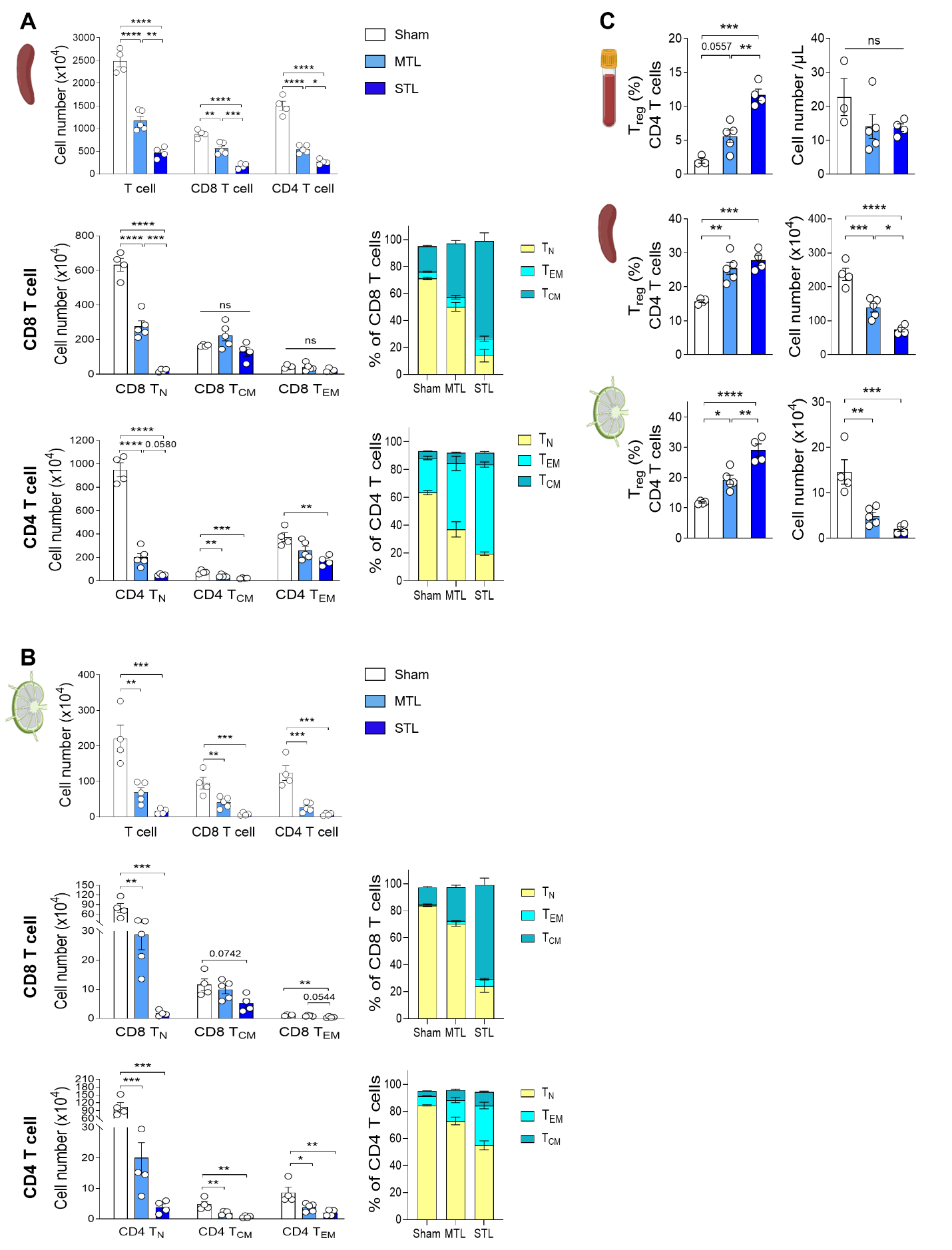
Supplementary Figures**

**Supplementary Figure S1. Characterization of T cell composition in secondary lymphoid organs of T-lymphopenic mice.**

Sham, MTL, and STL mouse models were established, as shown in Fig. 1A. **(A, B)** Seven weeks after sham or thymectomy, spleen and inguinal lymph nodes (iLNs) were harvested from each mouse model, and cells were analyzed by flow cytometry, respectively (*n* = 4-5 per group). **(A)** Flow cytometric analysis of total, CD8, and CD4 T cells and their subpopulations in spleen. **(B)** Flow cytometric analysis of total, CD8, and CD4 T cells and their subpopulations in iLNs. **(C)** Percentage (left) and absolute number (right) of Foxp3^+^ T_reg_ cells in PB (top), spleen (middle), and iLNs (bottom). All the data are represented as means ± SEM and representative of three independent experiments. ^*^*P* < 0.05; ^**^*P* < 0.01; ^***^*P* < 0.001; and ^****^*P* < 0.0001, one-way ANOVA with Bonferroni’s multiple comparison test for **(A-C)**.
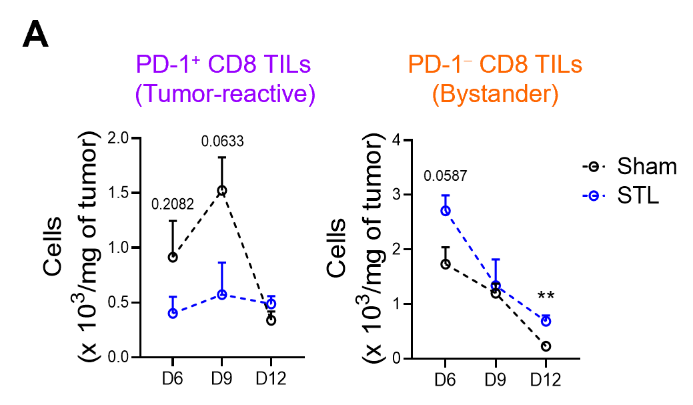


**Supplementary Figure S2. Absolute numbers of PD-1^+^ and PD-1^−^ CD8 TILs in sham and STL mice.**

Advanced MC38 tumor-bearing sham (*n* = 4 per group) and STL (*n* = 4 per group) mice were administered CCRT (D0). Tumor tissues were harvested from each mouse model, and cells were analyzed by flow cytometry at indicated days, as shown in Fig. 3A. **(A)** Absolute numbers of PD-1^+^ (purple) and PD-1^−^ (orange) CD8 TILs in sham and STL mice at indicated days. All the data are represented as means ± SEM. ^*^*P* < 0.05; ^**^*P* < 0.01; ^***^*P* < 0.001; and ^****^*P* < 0.0001, unpaired two-tailed Student’s t-test at the same time point **(A)**.


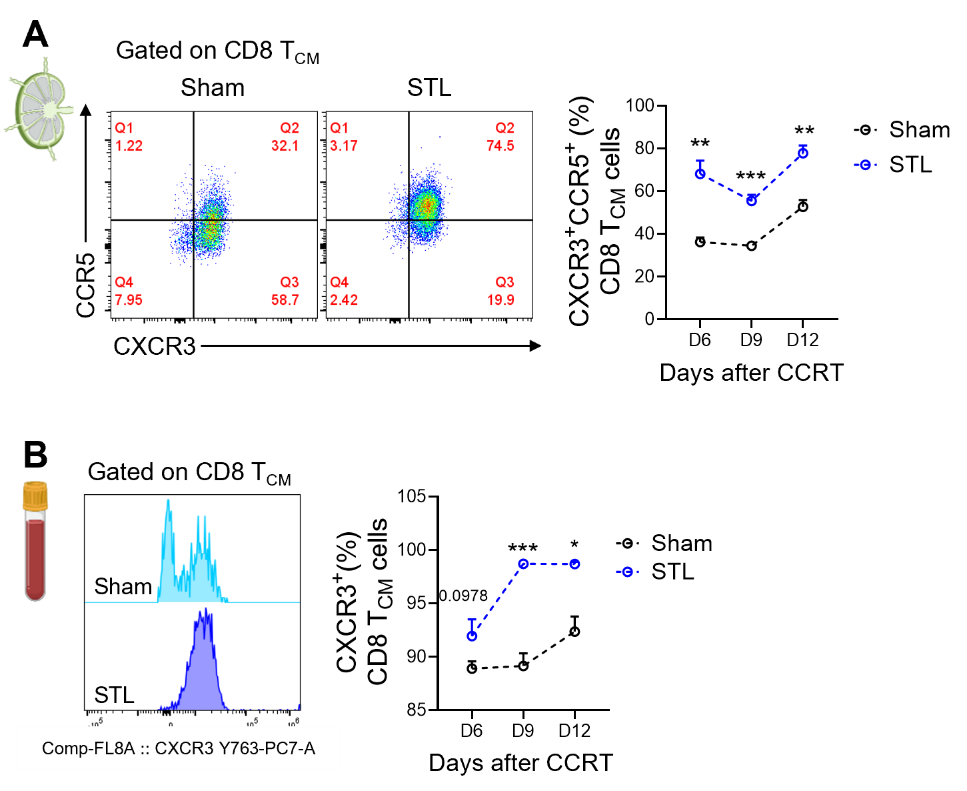


**Supplementary Figure S3. Chemokine receptor expression on peripheral CD8 T_CM_ cells increases in T-lymphopenic conditions.**

Advanced MC38 tumor-bearing sham (*n* = 4-5 per group) and STL (*n* = 3-4 per group) mice were administered CCRT (D0). TDLNs and PB were harvested from each mouse model, and cells were analyzed by flow cytometry at indicated days, as shown in Fig. 3A. **(A)** Representative flow cytometry plots (left) and percentage (right) of CXCR3^+^CCR5^+^ cells among CD8 T_CM_ in TDLNs. **(B)** Representative histogram (left) and percentage (right) of CXCR3^+^ cells among CD8 T_CM_ cells in PB. All the data are represented as means ± SEM. ^*^*P* < 0.05; ^**^*P* < 0.01; ^***^*P* < 0.001; and ^****^*P* < 0.0001, unpaired two-tailed Student’s t-test at the same time point **(A, B)**.


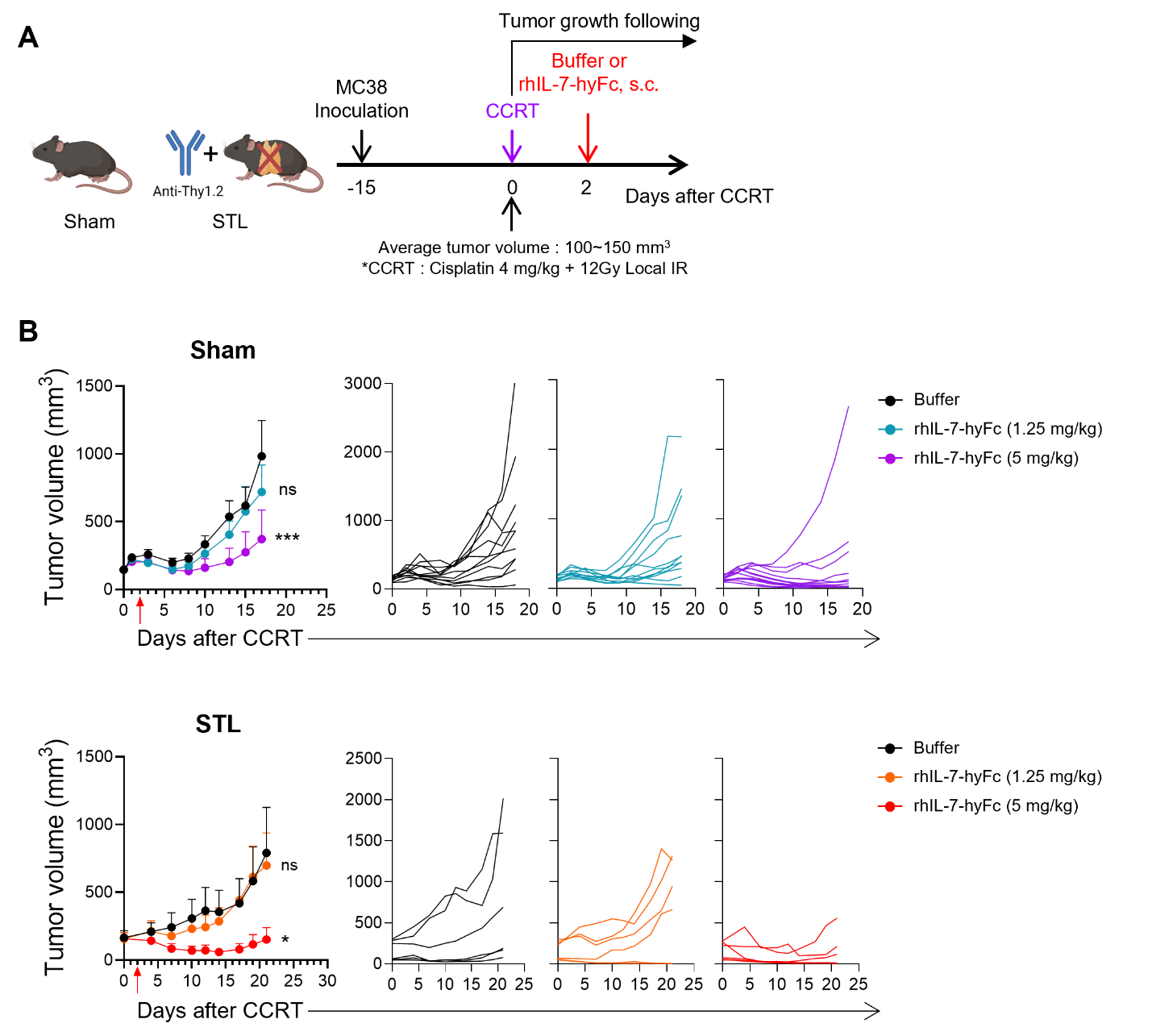


**Supplementary Figure S4. rhIL-7-hyFc dose escalation study in sham and T-lymphopenic mice with advanced MC38 tumors treated with CCRT.**

**(A)** Experimental scheme. Advanced MC38 tumor-bearing sham (n = 11-12 per group) and STL (n = 6 per group) mice were administered CCRT and treated with a single dose of rhIL-7-hyFc (1.25 or 5 mg/kg) or buffer via s.c. route 2 days after CCRT administration. **(B)** Mean tumor volumes (left) and individual tumor growth (right) for each group of sham (top) and STL (bottom) mice. ^*^*P* < 0.05; ^**^*P* < 0.01; ^***^*P* < 0.001; and ^****^*P* < 0.0001, two-way ANOVA with Bonferroni posttests (B and C).


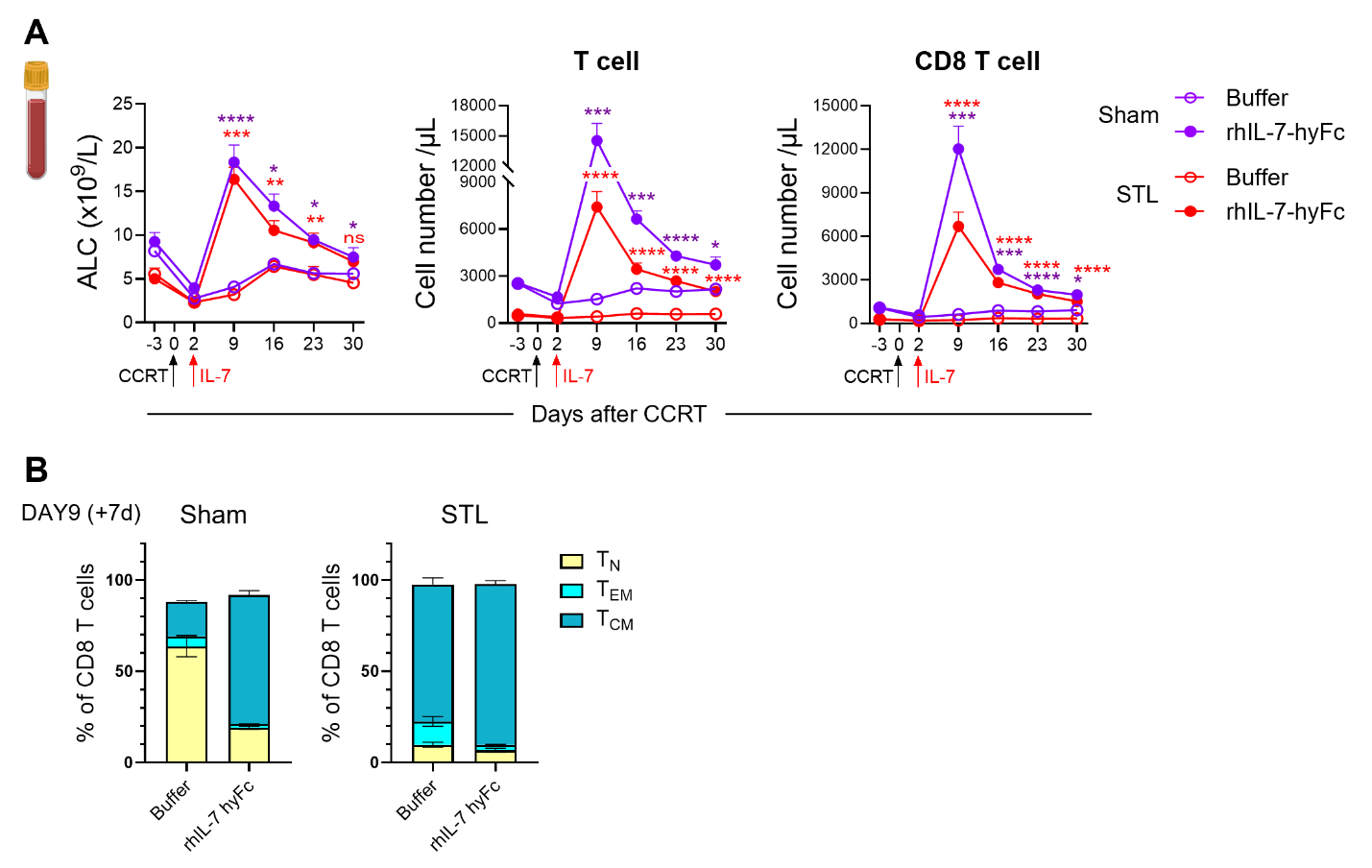


**Supplementary Figure S5. Peripheral CD8 T cell dynamics following rhIL-7-hyFc treatment in sham and T-lymphopenic mice.**

**(A, B)** Advanced MC38 tumor-bearing sham (*n* = 4 per group) and STL (*n* = 6 per group) mice were administered CCRT (D0) and treated with 5 mg/kg of rhIL-7-hyFc or buffer via s.c. route 2 days after CCRT administration (D2). PB was collected 5 days before and 0, 7, 14, 21, and 28 days after rhIL-7-hyFc or buffer treatment, and immune cells were analyzed by flow cytometry at indicated days (D-3, 2, 9, 16, 23, and 30). **(A)** Changes in peripheral blood ALC (left), total T cell (middle), and CD8 T cell count (right) from sham and STL mice at time points indicated in the graph. **(B)** Composition of CD8 T cell subsets in buffer or rhIL-7-hyFc-treated sham and STL mice at 7 days after drug treatment (D9). All the data are represented as means ± SEM and representative of two independent experiments. Statistical analysis was performed to compare the values between the buffer-treated versus rhIL-7-hyFc-treated group and the color of asterisks represents the comparison within sham (purple) and STL (red) mice. ^*^*P* < 0.05; ^**^*P* < 0.01; ^***^*P* < 0.001; and ^****^*P* < 0.0001, unpaired two-tailed Student’s t-test at the same time point **(A)**.


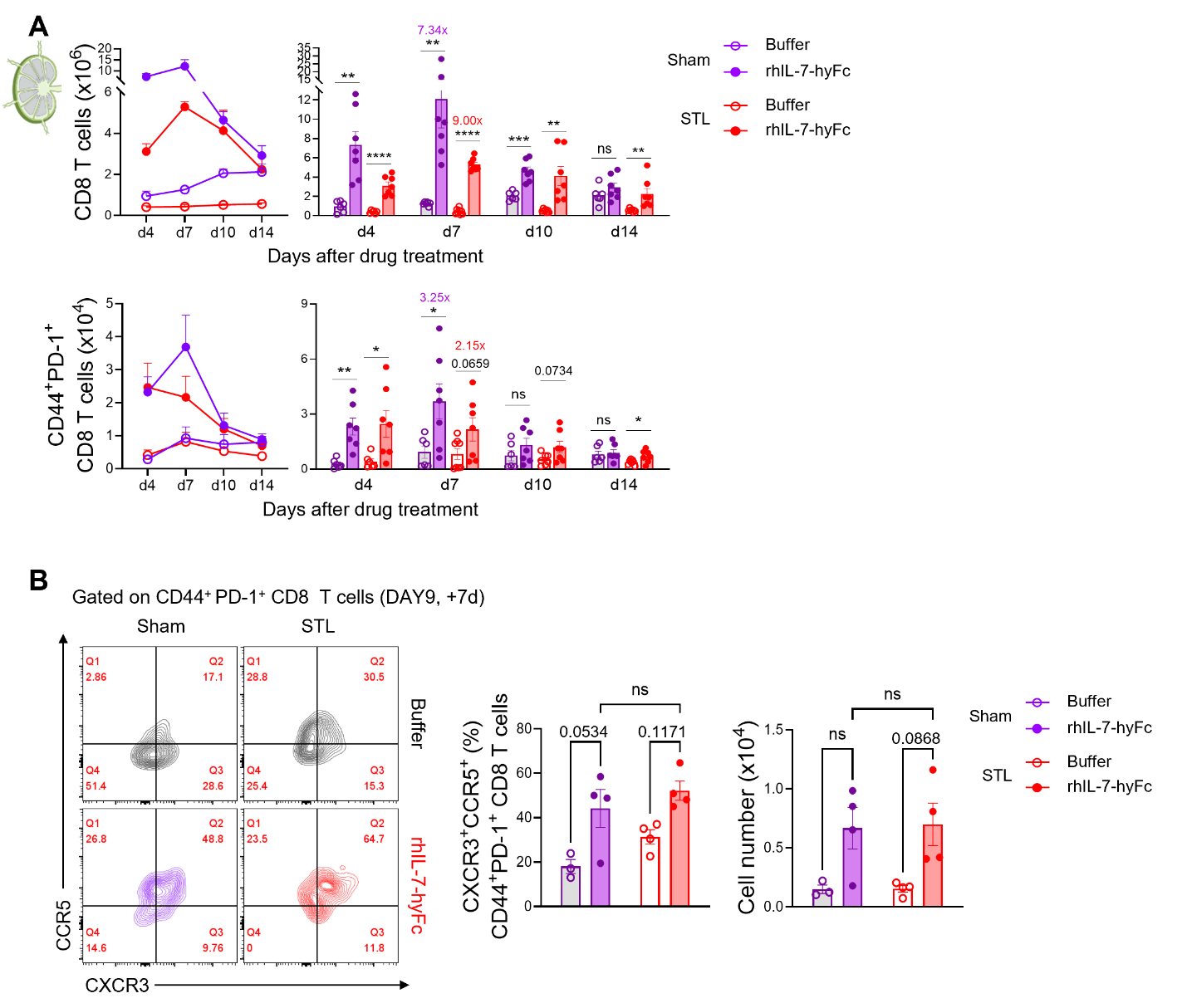


**Supplementary Figure S6. rhIL-7-hyFc expands PD-1^+^ CD8 T cells with enhanced chemokine receptor expression in the tumor-draining lymph nodes.**

**(A, B)** Advanced MC38 tumor-bearing sham or STL mice were treated, as shown in Fig. 4A. TDLNs were harvested from sham or STL mice at 4, 7, 10, and 14 days following rhIL-7-hyFc or buffer treatment, and immune cells were analyzed by flow cytometry at indicated time points. **(A)** Absolute numbers of total (top) and activated CD44^+^PD-1^+^ (bottom) CD8 T cells at indicated time points. **(B)** Representative flow cytometry plots for CD44^+^PD-1^+^ CD8 T cells expressing CXCR3 and CCR5 chemokine receptors from buffer or rhIL-7-hyFc-treated sham or STL mice. Bar graph displaying the proportion and number of CXCR3^+^CCR5^+^ cells among CD44^+^PD-1^+^ CD8 T cells in buffer-treated versus rhIL-7-hyFc-treated sham or STL mice at 7 days post-treatment. All the data are represented as means ± SEM of pooled biologically independent samples from two independent experiments (*n* = 3-4 per group) for **(A)**. ^*^*P* < 0.05; ^**^*P* < 0.01; ^***^*P* < 0.001; and ^****^*P* < 0.0001, unpaired two-tailed Student’s t-test at the same time point **(A)** and one-way ANOVA with Bonferroni’s multiple comparison test **(B)**.


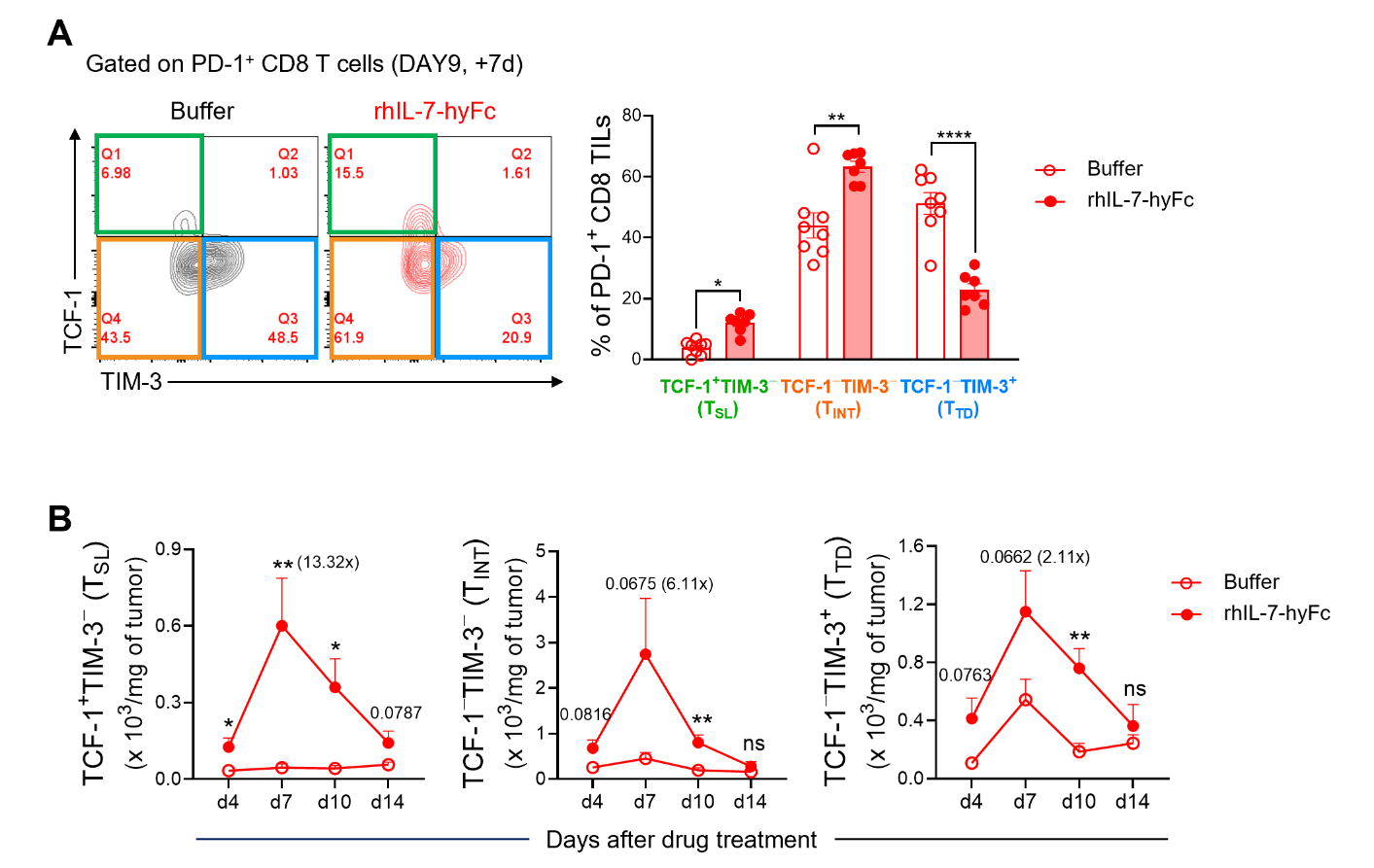


**Supplementary Figure S7. rhIL-7-hyFc preferentially expands TCF-1^+^TIM-3^−^ stem-like CD8 TILs.**

**(A, B)** Flow cytometric analysis of PD-1^+^ CD8 TILs following rhIL-7-hyFc or buffer treatment from CCRT-treated advanced MC38 tumor-bearing STL mice at indicated days, as shown in Fig. 4A. **(A)** Representative flow cytometry plots (left) and percentage (right) of PD-1^+^ CD8 TIL subpopulations in buffer treated versus rhIL-7-hyFc-treated STL mice at 7 days post-treatment. **(B)** Number of TCF-1^+^TIM-3^−^ (stem-like, T_SL_), TCF-1^−^TIM-3^−^ (intermediate exhausted, T_INT_), and TCF-1^−^TIM-3^+^ (terminally differentiated, T_TD_) CD8 TILs at indicated time points for indicated treatment groups. All the data are represented as means ± SEM of pooled biologically independent samples from two independent experiments (n = 3-4 per group) for **(A, B)**. ^*^*P* < 0.05; ^**^*P* < 0.01; ^***^*P* < 0.001; and ^****^*P* < 0.0001, unpaired two-tailed Student’s t-test at the same time point **(A, B)**.

**Supplemental references**

1. Feng JF, Liu JS, Huang Y. Lymphopenia predicts poor prognosis in patients with esophageal squamous cell carcinoma. Medicine (Baltimore). 2014;93(27):e257.

2. Kou F, Lu Z, Li J, Zhang X, Lu M, Zhou J, et al. Pretreatment lymphopenia is an easily detectable predictive and prognostic marker in patients with metastatic esophagus squamous cell carcinoma receiving first-line chemotherapy. Cancer Med. 2016;5(5):778-86.

3. Wu ES, Oduyebo T, Cobb LP, Cholakian D, Kong X, Fader AN, et al. Lymphopenia and its association with survival in patients with locally advanced cervical cancer. Gynecol Oncol. 2016;140(1):76-82.

4. Onal C, Yildirim BA, Guler OC, Mertsoylu H. The Utility of Pretreatment and Posttreatment Lymphopenia in Cervical Squamous Cell Carcinoma Patients Treated With Definitive Chemoradiotherapy. Int J Gynecol Cancer. 2018;28(8):1553-9.

5. Ray-Coquard I, Cropet C, Van Glabbeke M, Sebban C, Le Cesne A, Judson I, et al. Lymphopenia as a prognostic factor for overall survival in advanced carcinomas, sarcomas, and lymphomas. Cancer Res. 2009;69(13):5383-91.

6. Tredan O, Manuel M, Clapisson G, Bachelot T, Chabaud S, Bardin-dit-Courageot C, et al. Patients with metastatic breast cancer leading to CD4+ T cell lymphopaenia have poor outcome. Eur J Cancer. 2013;49(7):1673-82.

7. Suzuki R, Lin SH, Wei X, Allen PK, Welsh JW, Byers LA, Komaki R. Prognostic significance of pretreatment total lymphocyte count and neutrophil-to-lymphocyte ratio in extensive-stage small-cell lung cancer. Radiother Oncol. 2018;126(3):499-505.

8. Ceze N, Thibault G, Goujon G, Viguier J, Watier H, Dorval E, Lecomte T. Pre-treatment lymphopenia as a prognostic biomarker in colorectal cancer patients receiving chemotherapy. Cancer Chemother Pharmacol. 2011;68(5):1305-13.

9. Peron J, Cropet C, Tredan O, Bachelot T, Ray-Coquard I, Clapisson G, et al. CD4 lymphopenia to identify end-of-life metastatic cancer patients. Eur J Cancer. 2013;49(5):1080-9.

10. Deng W, Xu C, Liu A, van Rossum PSN, Deng W, Liao Z, et al. The relationship of lymphocyte recovery and prognosis of esophageal cancer patients with severe radiation-induced lymphopenia after chemoradiation therapy. Radiother Oncol. 2019;133:9-15.

11. Xu H, Lin M, Hu Y, Zhang L, Li Q, Zhu J, et al. Lymphopenia During Definitive Chemoradiotherapy in Esophageal Squamous Cell Carcinoma: Association with Dosimetric Parameters and Patient Outcomes. Oncologist. 2021;26(3):e425-e34.

12. Xu C, Jin JY, Zhang M, Liu A, Wang J, Mohan R, et al. The impact of the effective dose to immune cells on lymphopenia and survival of esophageal cancer after chemoradiotherapy. Radiother Oncol. 2020;146:180-6.

13. Davuluri R, Jiang W, Fang P, Xu C, Komaki R, Gomez DR, et al. Lymphocyte Nadir and Esophageal Cancer Survival Outcomes After Chemoradiation Therapy. Int J Radiat Oncol Biol Phys. 2017;99(1):128-35.

14. Zhao Q, Chen G, Ye L, Shi S, Du S, Zeng Z, He J. Treatment-duration is related to changes in peripheral lymphocyte counts during definitive radiotherapy for unresectable stage III NSCLC. Radiat Oncol. 2019;14(1):86.

15. Campian JL, Ye X, Brock M, Grossman SA. Treatment-related lymphopenia in patients with stage III non-small-cell lung cancer. Cancer Invest. 2013;31(3):183-8.

16. Cho O, Oh YT, Chun M, Noh OK, Lee HW. Radiation-related lymphopenia as a new prognostic factor in limited-stage small cell lung cancer. Tumour Biol. 2016;37(1):971-8.

17. Wang X, Lu J, Teng F, Yu J. Lymphopenia association with accelerated hyperfractionation and its effects on limited-stage small cell lung cancer patients' clinical outcomes. Ann Transl Med. 2019;7(16):385.

18. Dai D, Tian Q, Shui Y, Li J, Wei Q. The impact of radiation induced lymphopenia in the prognosis of head and neck cancer: A systematic review and meta-analysis. Radiother Oncol. 2022;168:28-36.

19. Byun HK, Kim N, Park S, Seong J. Acute severe lymphopenia by radiotherapy is associated with reduced overall survival in hepatocellular carcinoma. Strahlenther Onkol. 2019;195(11):1007-17.

20. Cho O, Oh YT, Chun M, Noh OK, Hoe JS, Kim H. Minimum absolute lymphocyte count during radiotherapy as a new prognostic factor for nasopharyngeal cancer. Head Neck. 2016;38 Suppl 1:E1061-7.

21. Liu LT, Chen QY, Tang LQ, Guo SS, Guo L, Mo HY, et al. The Prognostic Value of Treatment-Related Lymphopenia in Nasopharyngeal Carcinoma Patients. Cancer Res Treat. 2018;50(1):19-29.

22. Grossman SA, Ellsworth S, Campian J, Wild AT, Herman JM, Laheru D, et al. Survival in Patients With Severe Lymphopenia Following Treatment With Radiation and Chemotherapy for Newly Diagnosed Solid Tumors. J Natl Compr Canc Netw. 2015;13(10):1225-31.

23. Grossman SA, Ye X, Lesser G, Sloan A, Carraway H, Desideri S, et al. Immunosuppression in patients with high-grade gliomas treated with radiation and temozolomide. Clin Cancer Res. 2011;17(16):5473-80.

24. Wild AT, Ye X, Ellsworth SG, Smith JA, Narang AK, Garg T, et al. The Association Between Chemoradiation-related Lymphopenia and Clinical Outcomes in Patients With Locally Advanced Pancreatic Adenocarcinoma. Am J Clin Oncol. 2015;38(3):259-65.

25. Lee BM, Byun HK, Seong J. Significance of lymphocyte recovery from treatment-related lymphopenia in locally advanced pancreatic cancer. Radiother Oncol. 2020;151:82-7.

26. Cho Y, Park S, Byun HK, Lee CG, Cho J, Hong MH, et al. Impact of Treatment-Related Lymphopenia on Immunotherapy for Advanced Non-Small Cell Lung Cancer. Int J Radiat Oncol Biol Phys. 2019;105(5):1065-73.

27. Pike LRG, Bang A, Mahal BA, Taylor A, Krishnan M, Spektor A, et al. The Impact of Radiation Therapy on Lymphocyte Count and Survival in Metastatic Cancer Patients Receiving PD-1 Immune Checkpoint Inhibitors. Int J Radiat Oncol Biol Phys. 2019;103(1):142-51.

28. Ottonello S, Genova C, Cossu I, Fontana V, Rijavec E, Rossi G, et al. Association Between Response to Nivolumab Treatment and Peripheral Blood Lymphocyte Subsets in Patients With Non-small Cell Lung Cancer. Front Immunol. 2020;11:125.

29. Tomsitz D, Schlaak M, Zierold S, Pesch G, Schulz TU, Muller G, et al. Development of Lymphopenia during Therapy with Immune Checkpoint Inhibitors Is Associated with Poor Outcome in Metastatic Cutaneous Melanoma. Cancers (Basel). 2022;14(13).

30. Li S, Zou J, Liu C, Jiao X, Gong J, Li J, et al. Baseline derived neutrophil-to-lymphocyte ratio as a prognostic biomarker for non-colorectal gastrointestinal cancer patients treated with immune checkpoint blockade. Clin Immunol. 2020;212:108345.

31. Jing W, Xu T, Wu L, Lopez PB, Grassberger C, Ellsworth SG, et al. Severe Radiation-Induced Lymphopenia Attenuates the Benefit of Durvalumab After Concurrent Chemoradiotherapy for NSCLC. JTO Clin Res Rep. 2022;3(9):100391.

32. Friedes C, Chakrabarti T, Olson S, Prichett L, Brahmer JR, Forde PM, et al. Association of severe lymphopenia and disease progression in unresectable locally advanced non-small cell lung cancer treated with definitive chemoradiation and immunotherapy. Lung Cancer. 2021;154:36-43.

33. Ho WJ, Yarchoan M, Hopkins A, Mehra R, Grossman S, Kang H. Association between pretreatment lymphocyte count and response to PD1 inhibitors in head and neck squamous cell carcinomas. J Immunother Cancer. 2018;6(1):84.

34. Park JC, Durbeck J, Clark JR. Predictive value of peripheral lymphocyte counts for immune checkpoint inhibitor efficacy in advanced head and neck squamous cell carcinoma. Mol Clin Oncol. 2020;13(6):87.

35. Yin T, Wang P, Yu J, Teng F. Treatment-related lymphopenia impairs the treatment response of anti-PD-1 therapy in esophageal squamous cell carcinoma. Int Immunopharmacol. 2022;106:108623.
